# High-Speed Sequential DNA Computing Using a Solid-State DNA Origami Register

**DOI:** 10.1021/acscentsci.4c01557

**Published:** 2024-12-11

**Authors:** Qian Zhang, Mingqiang Li, Yuqing Tang, Jinyan Zhang, Chenyun Sun, Yaya Hao, Jianing Cheng, Xiaodong Xie, Sisi Jia, Hui Lv, Fei Wang, Chunhai Fan

**Affiliations:** † School of Chemistry and Chemical Engineering, New Cornerstone Science Laboratory, Frontiers Science Center for Transformative Molecules, National Center for Translational Medicine, Shanghai Jiao Tong University, Shanghai, 200240, China; ‡ Zhangjiang Laboratory, Shanghai, 201210, China

## Abstract

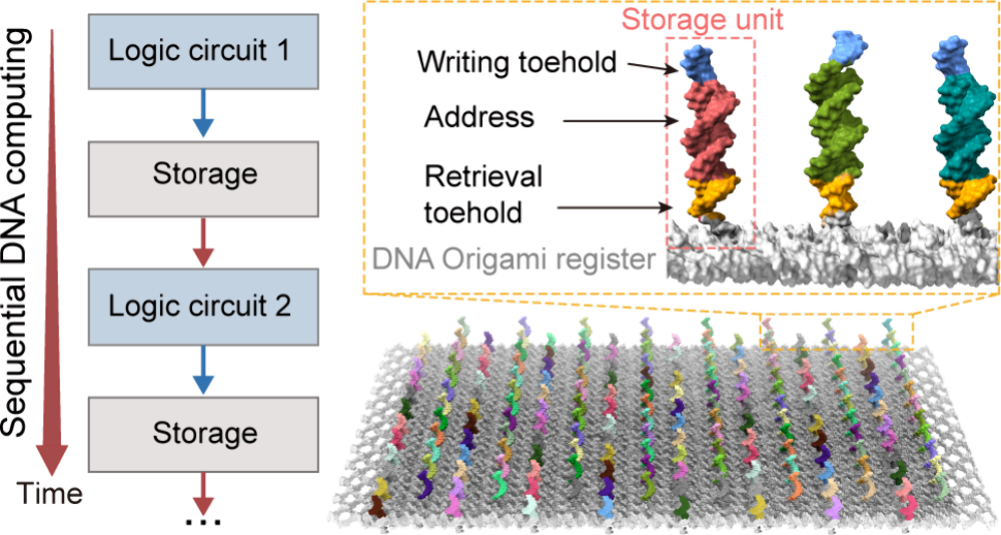

DNA computing leverages molecular reactions to achieve diverse information processing functions. Recently developed DNA origami registers, which could be integrated with DNA computing circuits, allow signal transmission between these circuits, enabling DNA circuits to perform complex tasks in a sequential manner, thereby enhancing the programming space and compatibility with various biomolecules of DNA computing. However, these registers support only single-write operations, and the signal transfer involves cumbersome and time-consuming register movements, limiting the speed of sequential computing. Here, we designed a solid-state DNA origami register that compresses output data from a 3D solution to a 2D surface, establishing a rewritable register suitable for solid-state storage. We developed a heterogeneous integration architecture of liquid-state circuits and solid-state registers, reducing the register-mediated signal transfer time between circuits to less than 1 h, thereby achieving fast sequential DNA computing. Furthermore, we designed a trace signal amplifier to read surface-stored signals back into solution. This compact approach not only enhances the speed of sequential DNA computing but also lays the foundation for the visual debugging and automated execution of DNA molecular algorithms.

## Abstract 2

This work developed a solid-state DNA origami register, with which a heterogeneous integration architecture is established and high-speed sequential DNA computing is demonstrated.

## Introduction

DNA molecules, characterized by their precise Watson–Crick base pairing, have been utilized to implement computing circuits by programming molecular interactions.^1^ Diverse functions and applications have been developed using DNA molecular computing, including logic circuits, neural network computing, medical diagnosis.^2−22^ Sequential computing can enhance information processing capabilities within a specific space. For instance, cells regulate complex life activities through sequential expression of genes;^23^ similarly, the operation of electronic computers also relies on sequential computing. Therefore, developing sequential DNA computing is essential for complex information processing with molecular reactions.

Sequential functions based-on DNA reactions such as timers,^24−26^ oscillators,^6,27,28^ and state machines^29^ have been demonstrated. However, these systems lack the capability of complex digital computing. In our previous work, we proposed a DPGA (DNA-based programmable gate array) with general-purpose programming capabilities.^20^ Through signal transmission mediated by DNA origami registers between DGPAs, asynchronous integration of DNA circuits, i.e., sequential computing, was realized. However, the data writing and reading on DNA origami registers require time-consuming procedures related to PEG precipitation or magnetic bead (MB)-mediated transfer, which typically takes more than several hours.^20^ This makes information transmission between circuits the rate-limiting step in sequential DNA computing.

**Figure 1:**
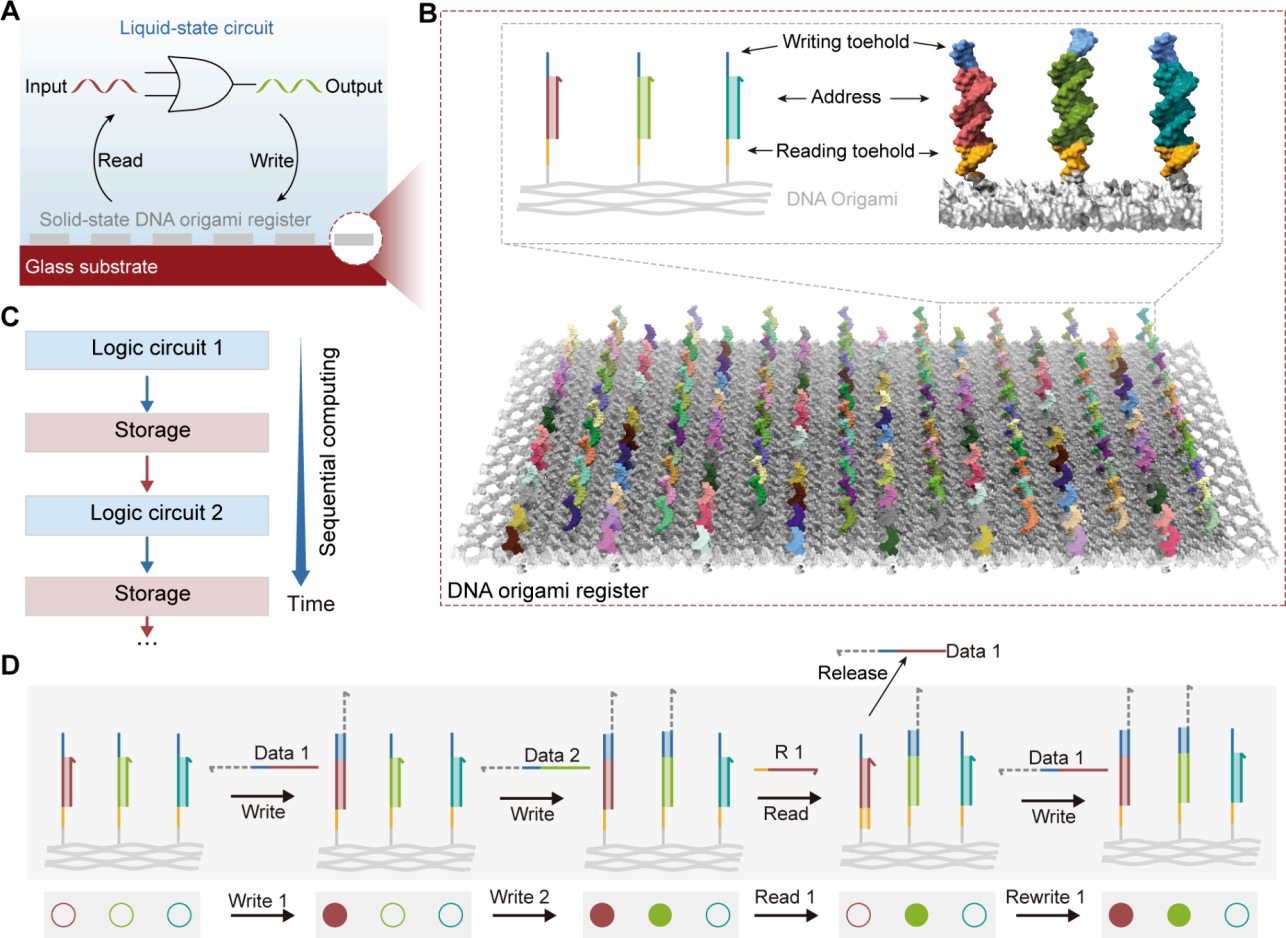
Heterogenous integration system for sequential DNA computing. (A) Schematic illustration of heterogeneous integration of liquid-state circuit and solid-state DNA origami register. (B) Molecular details of DNA origami register capable of working on a surface. About two hundred storage units can be constructed on an individual DNA origami by assigning specific sequences. Gray box shows molecular details of a storage unit. (C) Sequential computing can be realized by data exchange between a liquid-state circuit and a solid-state DNA origami register. (D) Addressable and rewritable data storage can be realized via writing toehold- and reading toehold-mediated strand displacement.

Here, we propose a heterogeneous integration architecture of liquid-state circuit and solid-state DNA origami register (Figure 1). By resetting the state of fixed DNA origami registers instead of moving them, we construct compact sequential digital circuits. Using rewritable DNA origami registers attached on a glass substrate, the output circuit in 3D solution is compressed down to a 2D surface by reducing its distribution dimension. After updating the circuit, the data stored on the surface are read back into the solution. DNA origami registers remain immobile throughout the entire process without the need for movement operations, thereby realizing a high-speed transmission in sequential DNA circuits.

## Results

### Design and Construction of Solid-State DNA Origami Register

We first designed a solid-state DNA origami register that operated on a glass substrate (Figure 1A). An individual DNA origami register can offer about 200 addressable storage units.^30,31^ Each storage unit contains three domains: address, writing toehold, and reading toehold (Figure 1B). DNA data can be written and stored in the solid-state DNA origami register by binding to a specific storage unit through the writing toehold-mediated strand displacement reaction (SDR, Figure 1C,D). When data need to be read out, a specific R molecule (e.g., R 1 for Data 1) replaces and releases the data strand by reading the toehold-meditated SDR, allowing it to participate in the circuit in solution. Along with the data being read out, the storage unit is reset, allowing for the corresponding data to be written again. This rewritable DNA origami register eliminates the need for change registers during sequential computing, thereby removing the requirement for moving registers.

**Figure 2:**
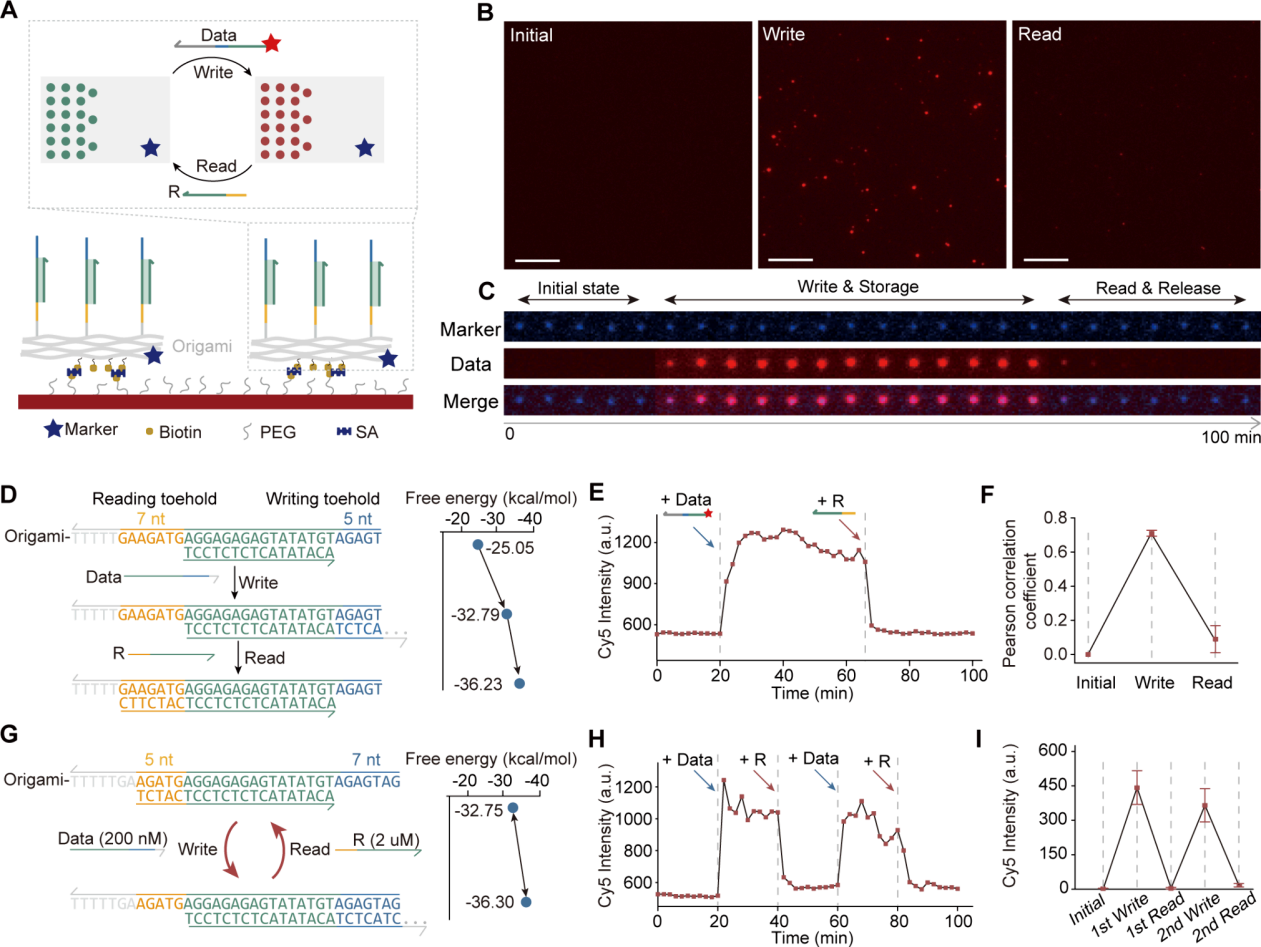
Engineering solid-state DNA origami registers for fast and efficient data storage on a surface. (A) Schematic diagram showing in situ monitoring data writing and reading of a solid-state DNA origami register. (B) Single-molecule fluorescence images showing fluorescence changes during the data writing and reading processes. (C) Snapshots of an individual origami register at the indicated time points, with the blue channel showing the fluorescence ATTO-488 marker and the red channel showing the fluorescence of Cy5-labeled data strands. (D) Molecular details and changes in free energy for a single-time DNA origami register. (E) A representative trace depicting the changes in the Cy5 fluorescence signal on a single DNA origami register during the writing and reading of the Data molecules. (F) Changes in colocalization of marker and Data before and after writing and reading information on the DNA origami register. Data are mean ± SD, *n* = 3. (G) Molecular details and changes in free energy of a rewritable DNA origami register. (H) A representative trace depicting changes in a Cy5 fluorescence signal on a single DNA memory during two cycles of writing and reading the data strands. (I) Changes in the Cy5 fluorescence intensity during two cycles of data writing and reading. Error bars represent SD, *n* = 3.

Next, we experimentally implemented the solid-state DNA origami register (Figure 2A). To verify the data writing and reading capabilities of solid-state storage, we first tested and observed in real-time the data writing and reading processes using a DNA origami register containing one storage region. Labeling the data strand with a Cy5 fluorescent molecule, we employed the total internal reflection fluorescence microscopy (TIRFM) to monitor the changes in fluorescence signals on each individual DNA origami register. The marker region of the origami register was labeled with ATTO488 molecules for localization (Figures S1 and S2). We first examined the DNA origami registers that have been prewritten with data strands to validate the feasibility of single-molecule observation. Through dual-channel fluorescence imaging, we observed distinct colocalization of data and marker signals (Figure S3). Subsequently, DNA origami registers in the initial state were immobilized onto the surface. Minimal fluorescence signals were detected in the absence of the input data strands in the Cy5 signal channel (Figures 2B and S3–S4). Upon the addition of labeled data strands, distinct fluorescent spots emerged on the locations of DNA origami registers, confirming that data strands were successfully written. Then, the Cy5 fluorescent spots on the surface disappeared after the addition of complementary R strands, showing the release of data strands from DNA origami registers. Meanwhile, by real-time monitoring the changes in fluorescence intensity of each DNA origami register, we observed the entire dynamic process of signal storage and readout (Figure 2C).

The single read-write DNA origami register is based on free-energy-driven reactions (Figure 2D), with the writing toehold (7 nt) being longer than the reading toehold (5 nt). Both data writing and reading operations result in lower free energy, ensuring high writing and reading efficiency. Fluorescence intensity trajectory from a single DNA origami register showed that the data writing process was completed within 10 min as the fluorescence intensity reached a plateau, and the reading process was completed in 5 min after the readout command, returning to a fluorescence-free state (Figure 2E). Comparing fluorescence colocalization probability (Figure 2F) across all DNA origami registers in the field of view, we observed a significant increase in fluorescence intensity for each register after data writing and a drop back to the background level after data reading. This indicates that conventional liquid-state DNA origami registers on the surface still support efficient data writing and reading and exhibit a rapid response.

When the writing toehold is shorter than the reading toehold, the binding of the R strand results in a more stable structure, making it difficult for the data to be written again. Therefore, we attempted to use a longer writing toehold combined with a shorter reading toehold to optimize the ability for repeated data writing. Subsequently, we increased the concentration of the R strand to read the stored information and reset the storage unit (Figure 2G). To verify this, we conducted repeated writing and reading experiments and observed similar fluorescence changes with each cycle of data writing and reading (Figure 2H and Figure S4A). Through optimization of the lengths of both toeholds of extended strands (*L*_Writing_ = 7 nt, *L*_Reading_ = 5 nt) and the concentration of R strand (1–2 μM), the efficiency of data rewriting could exceed 80% (Figures 2I and S5).

### Heterogeneous Integration of Circuit and Storage Using a Solid-State DNA Origami Register

**Figure 3:**
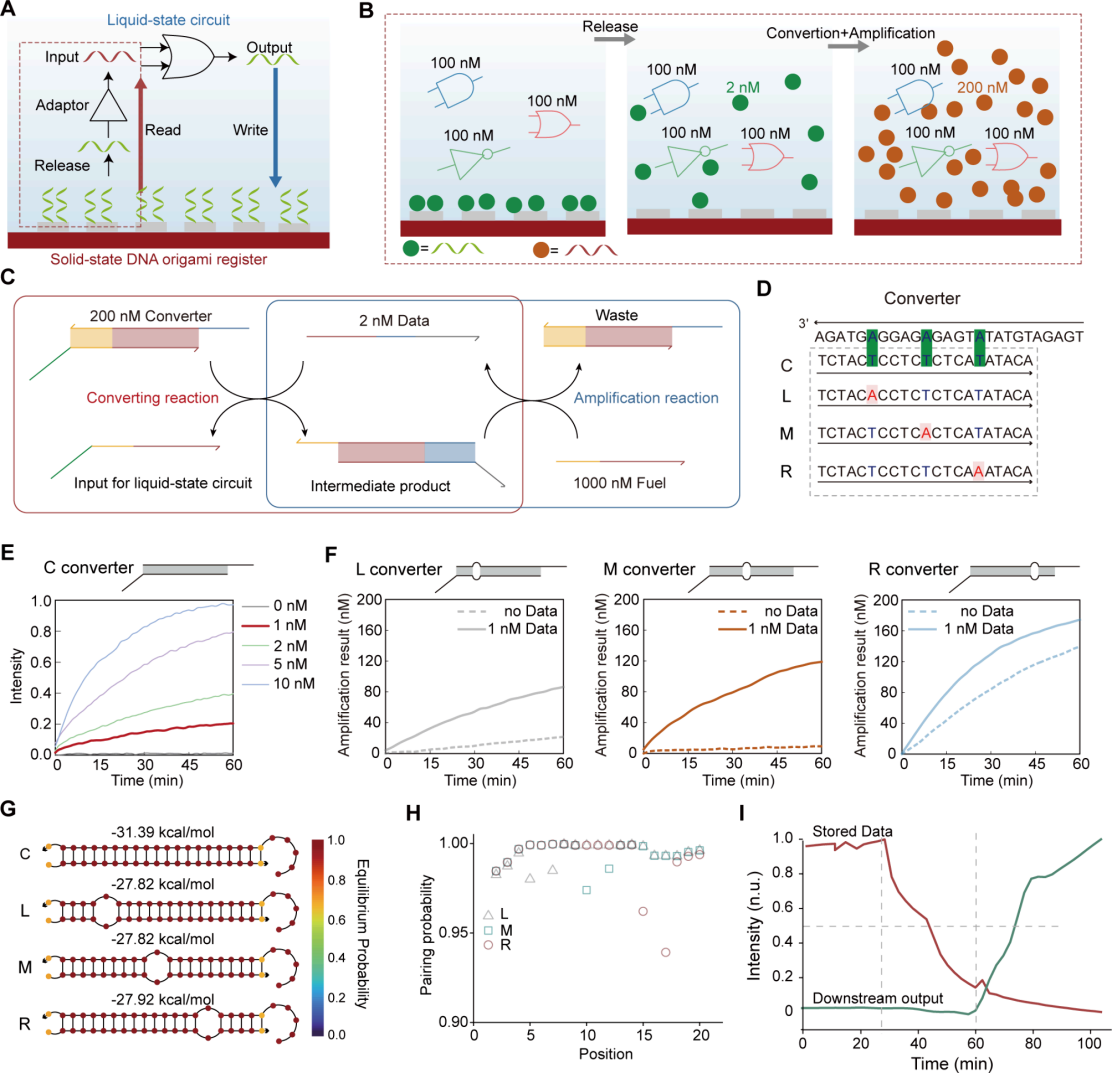
Heterogeneous integration of circuit and storage with an optimized surface-to-solution adaptor. (A) Working principle of the heterogeneous integration system. (B) Signal tranformation processes required to read out the output signal from the solid-state register to a liquid-state circuit. (C) Molecular implementation and chemical reaction network of adaptor. The red box represents the data converting reactions, and the blue box signifies the amplification reactions. (D) Sequences of the binding regions of the four converters; mismatched positions are indicated in red. (E) Fluorescence kinetics of adaptor with the C Converter of Data signals at 0, 1, 2, 5, and 10 nM. (F) Fluorescence kinetics of adaptors with L, M, and R converters with 1 nM Data. (G) Simulated free energy and equilibrium probability of C, L, M, and R converters. (H) Pairing probability along the hybridization region for L, M, and R converters. (I) Signal changes during the process of downstream circuit receiving the stored data from the solid-state register and writing the computing result back to the register.

Having established a solid-state DNA origami register, we next set out to develop a heterogeneous integration architecture (Figure 3A). The integration of the liquid-state circuit and solid-state register requires that the output from the liquid-state circuit can be written into the register and that data stored in the solid-state register can be read out and participate in the subsequent computing (Figure 3A). The process of writing the output from the solution into the solid-state register has been validated. Reading the output data stored in the solid-state register and converting it into input for the downstream circuit is key to achieving heterogeneous integration.

Reading data from the solid-state DNA origami register into solution results in an expanded distribution dimension. Consequently, even a very high surface density generates a low signal concentration in 3D space (∼1 nM in our system, see Materials and Methods). Concentration matching between the surface (1 nM) and solution (100 nM) is therefore required to support fast computations in solution. Therefore, we developed an adapter that can receive a trace DNA signal, converting and amplifying it into a high-concentration input signal (Figure 3B). The adapter consists of two functional modules: converter and amplifier (Figure 3C). The Data strand bound to the Converter to initiate strand displacement, generating an intermediate product and an Input for the downstream circuit. In the amplifier module, the Fuel strand binds to the intermediate product, releasing the Data strand, allowing it to participate in the converter module and replace another Input. Through cycles of consuming Data to generate Input and then restoring Data, one Data strand can generate multiple Input strands, thereby achieving signal amplification. The functionality of the amplification reaction was verified by observing the fluorescence recovery with a quenched reporter (Figure S6).

When utilizing conventional converters reliant on reversible strand displacement,^3,20,32−34^ achieving effective amplification poses challenges when the signal concentration is below 10 nM (Figure 3E). Therefore, a signal amplifier that can quickly amplify trace signals is needed. Here we propose to accelerate the amplification reaction by engineering free energy and conformation of DNA molecules. Based on this, we conducted structural optimization of the molecular design of the Converter molecule by introducing a mismatch at three distinct positions (L, M, and R, Figure 3D). We tested the signal amplification performance using these three converters and found that the amplification speed for the input signal at 1 nM was all improved compared to the C Converter. Notably, the M structure demonstrated the most favorable signal-to-noise ratio among the three structures (Figure 3F). Despite the R Converter exhibiting the fastest amplification, its leakage rates were significantly higher (Figures 3E and S7–S8). NUPACK simulations^4,35^ showcased that introducing 1 bp mismatches at these positions reduced the overall free energy of the structure, consistent with the observed increase in the amplification rate (Figure 3G). Further scrutiny of the pairing probability within the hybridization region revealed significant disparities. The R Converter exhibited a considerably lower pairing probability at the mismatch compared to the other structures. Additionally, the pairing probability of the 3 bp at the end diminished, hinting at the potential for fuel strand substitution when absent of the data strand, thus contributing to increased leakage of the R structure (Figure 3F,H). Therefore, the M Converter was used to construct the adaptor.

We further utilized the M Converter to conduct reaction experiments with different concentrations of Data molecule and found that Data concentrations exceeding 2 nM resulted in reaction yields higher than 90% after 1 h. Notably, even with as low as 1 nM Data, the reaction yield reached approximately 60% and generated more than 100 nM input, meeting the required concentration and reaction rate for the solution reaction (Figure S9). With the optimized adaptor, we integrated a solid-state DNA origami register and a downstream switching circuit. Results showed that the output of the downstream circuit was generated and written into the register after the stored data was released for a certain period (Figure 3I), which indicates that after the data in the solid-state register are read out, it can be effectively amplified and involved in the downstream liquid-state circuit. The establishment of data interaction loops between the solid-state register and the liquid-state circuit indicates that the heterogeneous integration architecture can operate effectively.

### Sequential DNA Computing with the Heterogeneous Integration Architecture

**Figure 4:**
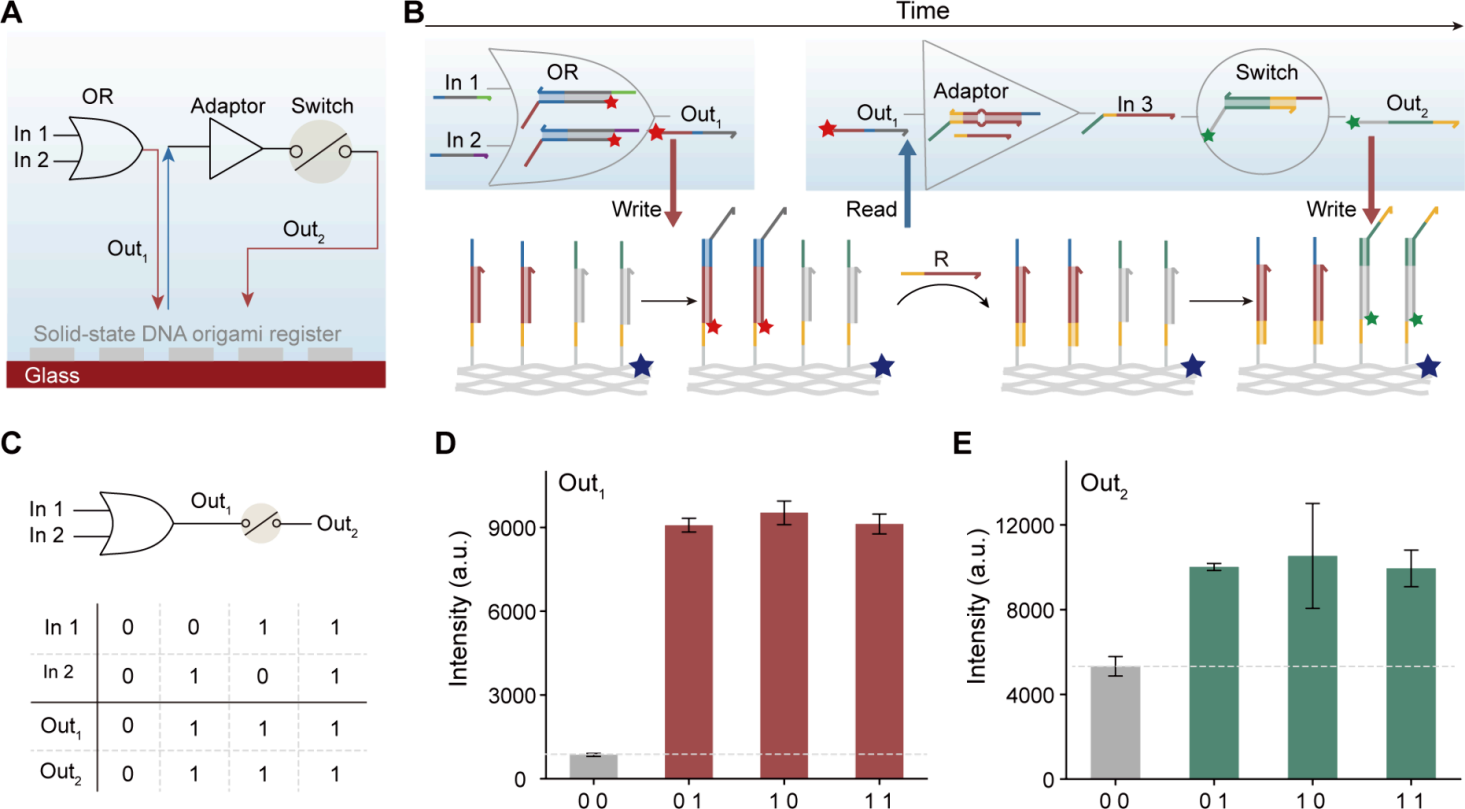
Sequential DNA computing with the heterogeneous integration system. (A and B) Schematic diagram (B) and molecular implementation (A) of a sequential circuit containing an OR gate and a switch. (C) Truth table of the OR-switch sequential circuit. (D) Operation results of OR circuit in the first layer. Error bars represent SD, *n* = 5. (E) Operation results of the whole circuit. Error bars represent SD, *n* = 5.

After optimizing the solid-state DNA origami register and the surface-to-solution adaptor, we further explored the use of this heterogeneous integration architecture for sequential computing. As a proof-of-concept, we tested the sequential cascading of an OR gate and a DNA switch mediated by solid-state storage (Figure 4A,B). In this setup, each DNA origami register comprises two distinct storage regions designated to store the outputs of the OR gate and Switch, respectively. The OR circuit was first run in solution, generating Out_1_ with a Cy5 fluorescent label. Out_1_ was then bound to the origami register, so that the result of the OR circuit could be quantified by the Cy5 fluorescence of the DNA origami. Then Out_1_ stored on the surface was released by R strands, thereby participating in the Switch function in the solution, generating output Out_2_ carrying a Cy3 fluorescent label. Figure 4A illustrates the changes in the fluorescent signals of DNA origami registered on the surface during the operation of the cascaded circuits. Experimental results showed the correct operation of the circuits for all four input combinations, demonstrating accurate functionality for both Out_1_ and Out_2_ (Figures 4C–E and S10–S11).

### Speed Improvement of Heterogeneous Integration-Based Sequential DNA Computing

**Figure 5:**
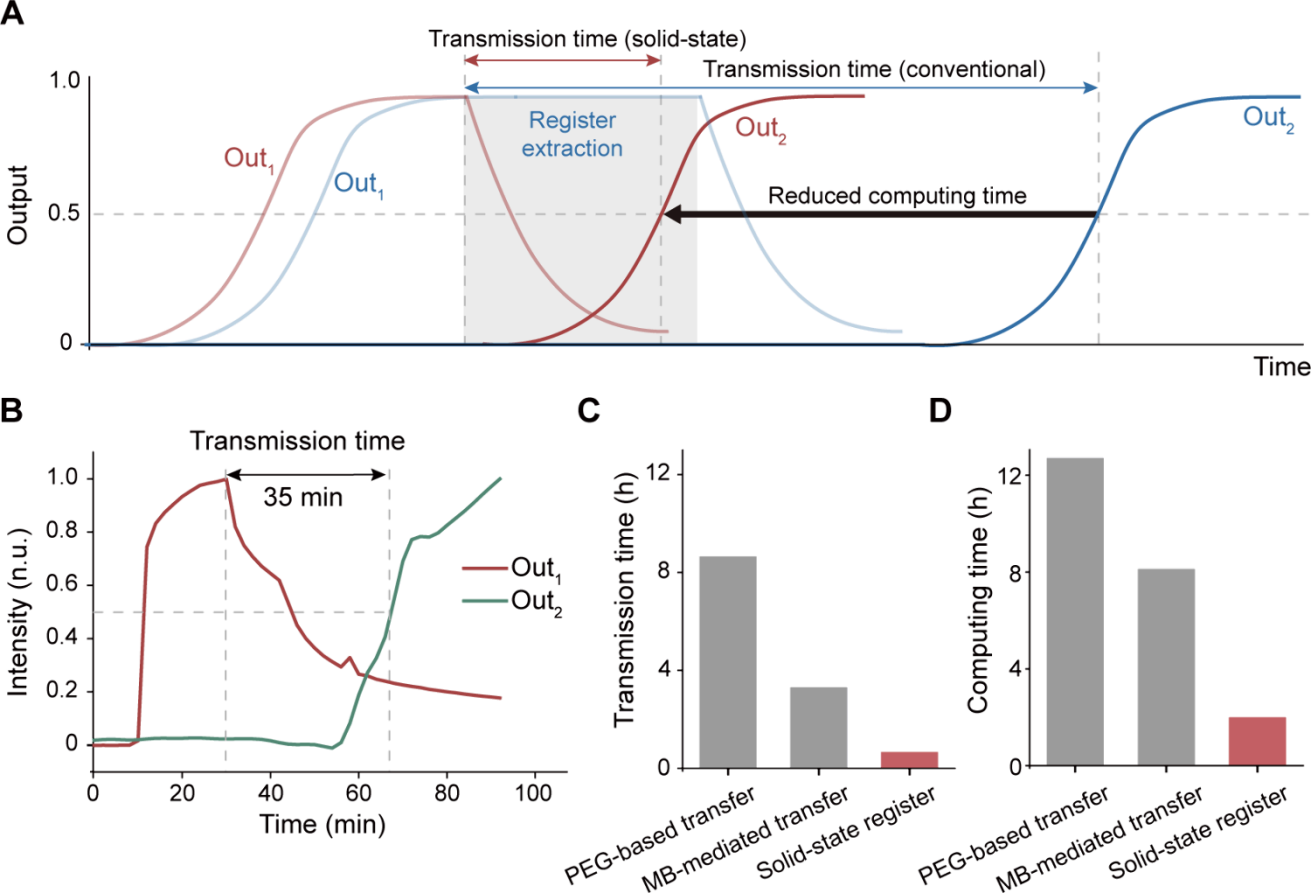
High-speed sequential computing with the heterogeneous integration approach. (A) Comparison of stages in sequential computing using solid-state and conventional DNA origami registers. (B) Dynamics of Out_1_ and Out_2_ signal intensity changes in the solid-state DNA origami register during circuit operation. (C) Comparison of transmission time for solid-state DNA origami register versus PEG- or MB-mediated transfer of registers. (D) Comparison of computing time for two-layered sequential computing.

When performing sequential computing using the heterogeneously integrated system, data transfer between circuits mediated by DNA origami register can be accomplished merely through spatial dimension transformations of output distribution. Compared to conventional liquid-state registers, this approach eliminated the register extraction step (Figure 5A), thereby reducing transmission time and improving the overall circuit operation speed. Analyzing the dynamics of the Out_1_ and Out_2_ signal changes on the register, we found that the downstream circuit generated the output within approximately 0.5 h after the reading command was added (Figure 5B). The signal transmission speed between circuits mediated by the solid-state register showed a remarkable improvement over systems where registers were needed to be moved (Figure 5C). The fast data transmission further enhanced the speed of sequential computing, with the entire computational process being completed within 1.5 h (Figure 5D).

## Discussion

In this work, we developed solid-state DNA storage with engineered rewritable DNA origami registers and established a heterogeneous integration architecture of a liquid-state circuit and solid-state DNA origami register. This approach significantly improved the signal transmission speed between sequentially cascaded circuits, enabling high-speed sequential DNA computing.

The immobilization and operation of the DNA origami register on the surface are crucial for achieving fast signal transmission. Data writing and reading operations can be accomplished merely through spatial dimension transformations. In contrast, the previous DPGA system used the DNA origami register as a carrier,^20^ moving it to transfer the stored data into the downstream circuit. The rapid signal amplification and conversion facilitated by the adapters also contributed to the improved computing speed. Through the optimization of the mismatch position, we elevated the free energy of the converter while maintaining structural stability and obtained adaptors with minimized leakage and a maximized amplification rate.

Here, we utilized single-molecule fluorescence imaging techniques to achieve real-time observation of data writing and reading processes on the solid-state origami register. While our primary focus was on constructing the solid-state origami register, where a data strand has multiple storage addresses, single-molecule sensitivity is not required for the current system.

Real-time imaging with single-molecule resolution can further enhance our understanding of address-related storage performance and holds great potential for single-copy data storage with improved information storage capacity in an individual DNA origami register. Such advancements would pave the way for more precise studies of molecular interactions and the functional dynamics of DNA-based systems, ultimately contributing to the development of more sophisticated DNA computing platforms.

This compact architecture not only enhances the speed of sequential DNA computing but also lays the foundation for the visual debugging and automated operation of DNA molecular algorithms. Fluorescent signals on the glass surface can be observed in real-time using TIRFM,^36−38^ allowing the surface-bound origami registers to temporarily store intermediate data from circuits and monitor the dynamics of molecular production and consumption during circuit operation. Compared with directly reading the final output, this method can facilitate the identification of abnormal gates. As DNA origami registers are immobilized on the glass substrate without the need for movement, sequential computing can be implemented merely by updating the circuit solution, making it easy to be integrated into microfluidic systems^39^ for further automation. Therefore, this strategy will lay the foundation for the development of compact DNA computers and nanomachines featuring intricate temporal functions.

## Materials and Methods

### Preparation of DNA Logic Circuits and DNA Origami Register

The DNA oligonucleotide strands were purchased from Sangon, GenScript, and Synbio Tech. The labeled DNA strands were dissolved in ultrapure water (Milli-Q), while the unlabeled DNA strands were dissolved in 1× TE buffer (Sangon). The total DNAs were quantified by measuring the absorbance peak at A260 nm using UV/vis spectrometry and stored at −20 °C. All dsDNA structures were formed through the annealing of corresponding ssDNA strands. The sequences corresponding to each structure are shown in Supplementary Tables S1–S8. To avoid erroneous structure formation, the annealing process was performed using a PCR thermal cycler (BioRad) for precise temperature control.

The thresholds and amplifiers used in the reactions were annealed in a 1:1.2 ratio, and the resulting dsDNAs were stored at a concentration of 100 μM. The reporters were annealed in a 1:1.5 ratio, with the strand labeling the quenching moiety in 50% excess compared to the fluorescently labeled strand. All experiments were performed in TE-Mg^2+^ buffer (10 mM Tris base, 1 mM EDTA, 12.5 mM MgCl_2_, pH 8.0), and the generated dsDNA was stored at 4 °C.

The register employed Rothemund’s strategy,^40^ assembling a rectangular DNA origami measuring 90 × 60 nm using M13m18 ssDNA (NEB), staple strands, extended staple strands, and complementary strands of extended staple strands in a 1:5:25:125 ratio for annealing. The experiments were conducted in 1× TAE-Mg^2+^ buffer (40 mM Tris base, 20 mM acetic acid, 2 mM EDTA, 12.5 mM magnesium acetate, pH 8.0) according to the previously described annealing procedure.^41^ After overnight stabilization at 4 °C, excess strands were removed by using polyethylene glycol (PEG) precipitation. Following a 1:1 ratio mixture with PEG buffer (15% PEG8000 (w/v), 5 mM Tris base, 1 mM EDTA, and 505 mM NaCl), the solution was centrifuged at low temperature (1000 rcf, 15 min, 4 °C), while maintaining a constant Mg ion concentration of 12.5 mM throughout the process. After the supernatant was removed, the pellet was dissolved in an appropriate amount of 1× TAE-Mg^2+^ buffer and mechanically mixed at 40 °C and 300 rpm for 12 h, and stored at 4 °C.

### AFM Imaging

The DNA rectangular origami was scanned and imaged by using the PeakForce mode of a Multimode VIII AFM system (Bruker, Inc.). Prior to scanning the sample with the peak force fluid tip, ∼40 μL of 1× TAE-Mg^2+^ buffer was added to the liquid cell to immerse the tip (ScanAsyst Fluid+). The scanned objects were purified with DNA origami registers. The 2 nM concentration of origami solution, with a volume of 10 μL, was deposited onto a freshly cleaved mica surface. After incubating for 5 min, AFM imaging was conducted.

### Fluorescence Kinetics Experiments

Real-time dynamic fluorescence detection was performed using a Synergy H1 Hybrid Multimode Reader (BioTek) and black 96-well assay plates (Corning). The fluorescence probe used in the experiment was a TET, with an excitation wavelength of 510 nm and an emission wavelength of 540 nm. The entire reaction process was carried out in 1× TE-Mg^2+^ buffer at a constant temperature of 18 °C ± 2 °C.

Initially, the premixed reaction solution without input was added to the assay plate, and the initial values were recorded as the baseline. Subsequently, the experiment was paused, and the input strands were added to the reaction solution. After thorough mixing, the assay plate was placed back in the reader to resume the experiment. The fluorescence kinetics of each reaction well were measured at intervals of 1–2 min.

The fluorescence intensity readings at each time point were normalized. The maximum level (output = 1) was determined by using the maximum fluorescence value observed during the experiment. The minimum level (output = 0) was defined as the lowest value obtained from all data at *t* = 0, and a threshold of 0.5 times the minimum value was used. Signals below 0.5 times the threshold were considered as 0, while signals above 0.5 times the threshold were considered as 1.

### Total Internal Reflection Microscopy (TIRF) Imaging Assays

The glass slides used in the experiments were treated prior to use.^42^ First, they were subjected to ultrasonic cleaning for 30 min each with deionized water (Milli-Q), acetone, and anhydrous ethanol. Subsequently, they were ultrasonicated for 1 h in 1 M KOH. After rinsing with deionized water and drying with nitrogen gas, the glass slides were silanized by treating the surface amino groups with a 1% (v/v) solution of silane reagent diluted in anhydrous ethanol for 1 h. The slides were then thoroughly rinsed with copious amounts of water, dried with nitrogen gas, and placed in an oven at 120 °C for 30 min. After removal from the oven and cooling to room temperature, the slides were stored in anhydrous ethanol.

Prior to the experiment, the glass slides were securely bonded to the reaction chambers by using double-sided tape. Sample barriers were placed on the surfaces of the glass slides to create reaction chambers. Subsequently, the reaction solution containing Biotin-PEG-NHS and PEG-NHS was prepared by diluting them with the 0.1 M sodium bicarbonate buffer at a ratio of 1:16, freshly prepared for immediate use. The mixed solution was applied to the chambers on the glass slides, allowing the bottom surface of the reaction chamber to react with biotin-PEG-NHS and PEG-NHS, thereby passivating and modifying the glass slide surface with Biotin and establishing fully functional reaction chambers for subsequent experiments.

At the beginning of the experiments, 1 mg/mL Streptavidin (Thermo) was added to the reaction chamber and incubated for 5 min, followed by washing off the excess protein. Then, purified and assembled DNA origami registers with Biotin modifications, ranging from 50 pM to 2 nM, were added to the reaction chamber and incubated for 10 min, followed by washing off the excess DNA origami registers. In single-molecule observation experiments, Cy5-labeled information strands at a concentration of 50 nM were added to the reaction chamber first and incubated for 20 min, followed by washing. Then, R strands at a concentration of 1 μM were added and incubated for an additional 20 min.

In fluorescence intensity observation experiments, a final concentration of 100 nM gate, 50 nM threshold, and 12.5 mM MgCl_2_ were mixed with TE buffer to a final volume of 98 μL in a 1.5 mL EP tube. After the mixture was mixed, 2 μL of 10 μM input was added. After incubating in the tube for 20 min, the mixture was added to the reaction chamber and incubated for an additional 20 min. Excess strands were then washed off using a 1× TAE-Mg solution. After waiting for 10 min, 10 μL of 2 μM R strands were added and incubated for 20 min, followed by the addition of the switch circuit (50 nM gate, 100 nM adaptor), and incubated for another 20 min. All reactions were conducted in 1× TAE-Mg^2+^ solution, maintaining a temperature of 18 °C ± 2 °C. And throughout the experiments, continuous imaging was performed with a 2 min interval.

All reactions were conducted using the TIRF mode of Leica LAS X. Imaging was performed using an EMCCD camera (Andor iXon Ultra 897) and solid-state 488, 561, and 638 nm lasers. Filters used for the ATTO 488 channel: Excitation 488/10, Dichroic 488, and Emission 525/50. Filters used for Cy3 channel: Excitation 561/10, Dichroic 575, Emission 600/50. Filters used for Cy5 channel: Excitation 635/20, Dichroic 640, and Emission 700/75. The exposure time was set to 100 ms to achieve a relatively high signal-to-noise ratio.

### Concentration Matching Requirements Calculation

For our experiment system, the bottom surface of the chamber was 7 mm × 7 mm, and the size of the DNA origami was 90 nm × 60 nm. Ideally, the surface of the chamber is completely covered with ∼10^10^ DNA origami registers, that is ∼0.015 pmol. Each origami register consists of 19 extended strand sites. Hence, under ideal situation, there was ∼0.3 pmol sites for the data strand wiring. And the volume of the reaction buffer in the chamber is 100 μL. So, the maximum data strand release concentration is ∼3 nM. However, considering the efficiency of writing and releasing, we sought to develop an adaptor that is able to work on 1–2 nM.

## Supporting Information Available

Additional discussions and experimental results, including structural details of DNA origami registers, single-molecule characterization experiments, DNA amplification circuit testing, converter kinetics experiments, and sequences of all DNA strands used in the experiments (PDF)
