# Supplementary material for: High-Speed Sequential DNA Computing Using a Solid-State DNA Origami Register
Source: ACS Cent Sci. 2024 Dec 11;10(12):2285–93. doi: 10.1021/acscentsci.4c01557 (PMC11672539; doi:10.1021/acscentsci.4c01557)
Supplement: Supplementary file 1 — oc4c01557_si_001.pdf [file oc4c01557_si_001.pdf]

# Supporting Information

## High-speed sequential DNA computing using a solid-state DNA origami register

Qian Zhang[1,3], Mingqiang Li[1,3], Yuqing Tang[1], Jinyan Zhang[1], Chenyun Sun[1], Yaya Hao[1], Jianing Cheng[1], Xiaodong Xie[1], Sisi Jia[2,*], Hui Lv[1,2,*], Fei Wang[1,*], Chunhai Fan[1,*]

[1] School of Chemistry and Chemical Engineering, New Cornerstone Science Laboratory, Frontiers Science Center for Transformative Molecules, National Center for Translational Medicine, Shanghai Jiao Tong University, Shanghai, 200240, China.

[2] Zhangjiang Laboratory, Shanghai, 201210, China.

[3]These authors contributed equally

* Corresponding author. E-mail: jiass@zjlab.ac.cn, lvhui92@sjtu.edu.cn, wangfeu@sjtu.edu.cn, fanchunhai@sjtu.edu.cn.

# Supporting Information

## Supporting texts

1. Rewriting capability of the DNA origami register

2. Stability of stored data strands

3. Stability of the DNA origami register on the glass surface

4. Selection principle of R strand concentration

## Supporting figures:

Figure S1. Distribution of data and fluorescence strands in the DNA origami register

Figure S2. Detailed design of DNA origami register.

Figure S3. The fluorescence images showing the colocalization between ATTO 488 and Cy5.

Figure S4. Single-molecule fluorescence images showing fluorescence changes during the data writing and reading processes.

Figure S5. Representative traces and analysis graphs of repeated writing and reading of data, while $L_{Writing}$ = 5 nt and $L_{Reading}$ = 7 nt.

Figure S6. Amplified dynamic fluorescence signal with the conventional amplifier.

Figure S7. Dynamic simulation analysis of strand replacement between different converter and input, simulation time trajectory (B) and distance distribution within simulation time (C).

Figure S8. Dynamic fluorescence signal of the L (A), M (B), and R (C) amplifiers.

Figure S9. Amplification results for different concentrations of Data using M converter. Control experiments involved the introduction of double-stranded DNA extending from the origami memory and the memory itself into the system, assessing whether their inclusion triggers any leakage.

Figure S10. The fluorescence images showing the fluorescence does not change before and after adding the labelling gate.

Figure S11. The fluorescence images showing the fluorescence does not change with all reaction components present except input.

**Supporting tables:**

Table S1. Sequence of M13mp18 scaffold

Table S2. Sequences of DNA origami register staples

Table S3. Sequences of staples at complementary to Cy5-data strands

Table S4. Sequences of staples at complementary to Cy3-data strands

Table S5. Sequences of staples at complementary to ATTO 488 localized strands

Table S6. Sequences of staples at sites complementary to biotin modification strands

Table S7. Sequences of strands with special labelling

Table S8. Sequences of input, R, converter and block

## Supporting texts

### 1. Rewriting capability of the DNA origami register

Concerning the replacement and release capabilities of the data strands from the origami surface, using the data from Fig 2, we fitted the results and observed a noticeable decrease in rewriting efficiency as the number of cycles increased. Specifically, when extended to 10 cycles, the signal is expected to decrease to 14.8%. Based on the signal amplification capacity of the adapters, the current reaction system for data replacement on the origami surface can support up to 5 cycles (~ 50%) of repetition.

| Number of cycles | 1 | 2 | 3 | 4 | 5 | 6 | 7 | 8 | 9 | 10 |
|---|---|---|---|---|---|---|---|---|---|---|
| Rewriting efficiency | 82.6% | 68.3% | 56.4% | 46.6% | 38.5% | 31.8% | 26.3% | 21.7% | 18.0% | 14.8% |

### 2. Stability of stored data strands

In our system, the data strands form a 23 bp hybrid region with the origami, with a Tm of 63 ℃, allowing for stable binding under the experimental conditions at 20 ℃. In our setup, the origami serves as a register rather than a non-volatile long-term memory. Within our observation period, the data remained well-attached to the origami surface. Long-term storage may result in a gradual release of data into the solution, so if signals need to be stored for extended periods before reading, further evaluation of the release rate and optimization of storage conditions may be necessary.

### 3. Stability of the DNA origami register on the glass surface

We labeled a 12-point pattern on the DNA origami and utilized stochastic optical reconstruction microscopy (STORM) to image the pattern on the origami surface. The relevant data is supplied as Appendix figure 1, which demonstrates that the DNA origami structures remain intact on the glass surface.

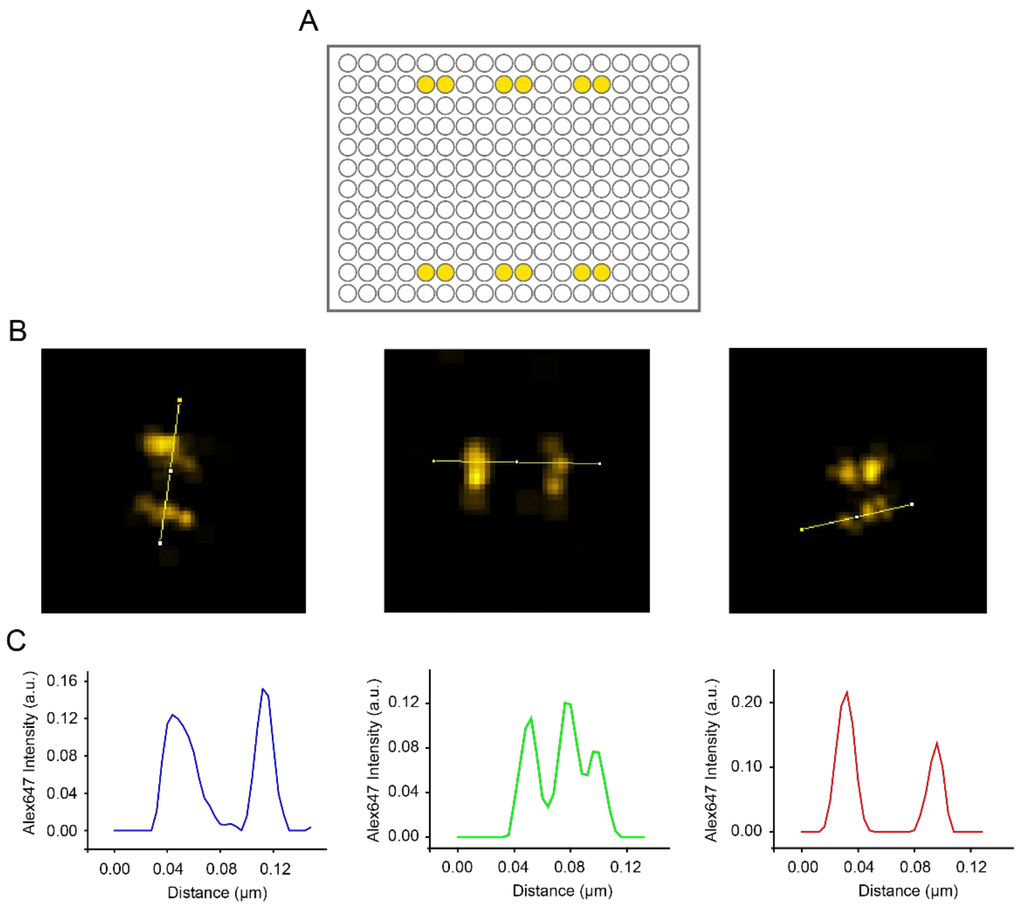


**Appendix figure 1. Imaging the pattern of the Alex647 in the DNA origami register on the glass surface using STORM.** (A) Distribution of Alex647 labelled strands on in the DNA origami register. (B) The fluorescence image of the Alex647 pattern showing the DNA origami register remaining intact. (C) The change of fluorescence intensity of Alex647 intensity curve along the drawn line.

## 4. Selection principle of R strand concentration

For single-use origami, both the writing and reading processes result in a decrease in free energy, so a slight excess of data and replacement strands ensures 100% strand "switch". Higher concentrations can further accelerate the reaction speed, and our 200 nM system can complete the signal switch within a few minutes.

For repeatedly writable registers, the signal reading process is associated with an increase in free energy, which requires increasing the concentration of DNA strands to control the reaction direction and speed it up. Given that the data capacity of the origami register at the interface is about 2 nM, 2 μM replacement strand allows for rapid and effective state transitions. We used

NUPACK to simulate the dependence of reading efficiency on replacement strand concentration, and the results show that the proportion of released data strands increases with higher replacement strand concentrations, achieving a reading efficiency of 96% with 2 μM of the replacement strand.

In fact, since the origami register is fixed to the surface of the reaction system, once the data strands are released, they are unlikely to rebind. Therefore, the actual reading efficiency of the solid-state register should be even higher than the results simulated by NUPACK.

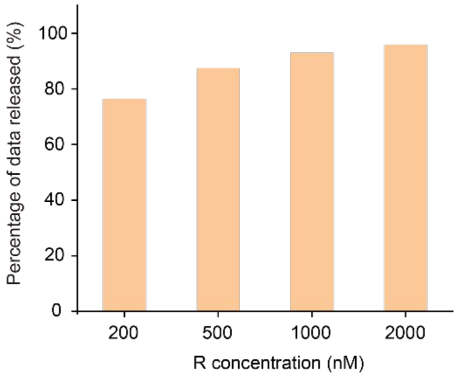


**Appendix figure 2. Data reading efficiency corresponding to different R strand concentrations based on NUPACK simulation.**

**SUPPORTING FIGURES**

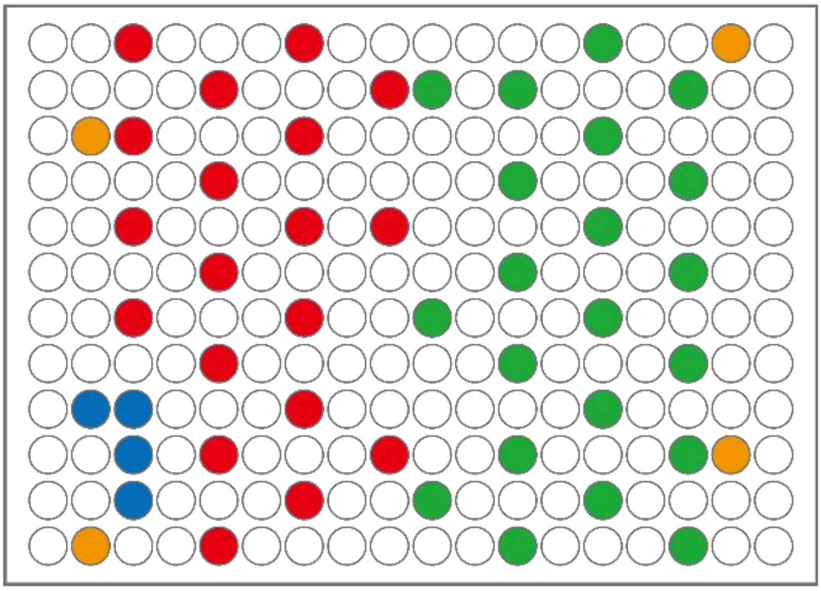

**Figure S1. Distribution of data and fluorescence strands in the DNA origami register.**

The red circles represent docking sites of the information strands labeled with Cy5, the green circles represent docking sites of the information strands labeled with Cy3, the orange circles represent docking points of the fixed strands labeled with Biotin, and the blue circles represent docking points of the indicator strands labeled with ATTO 488.

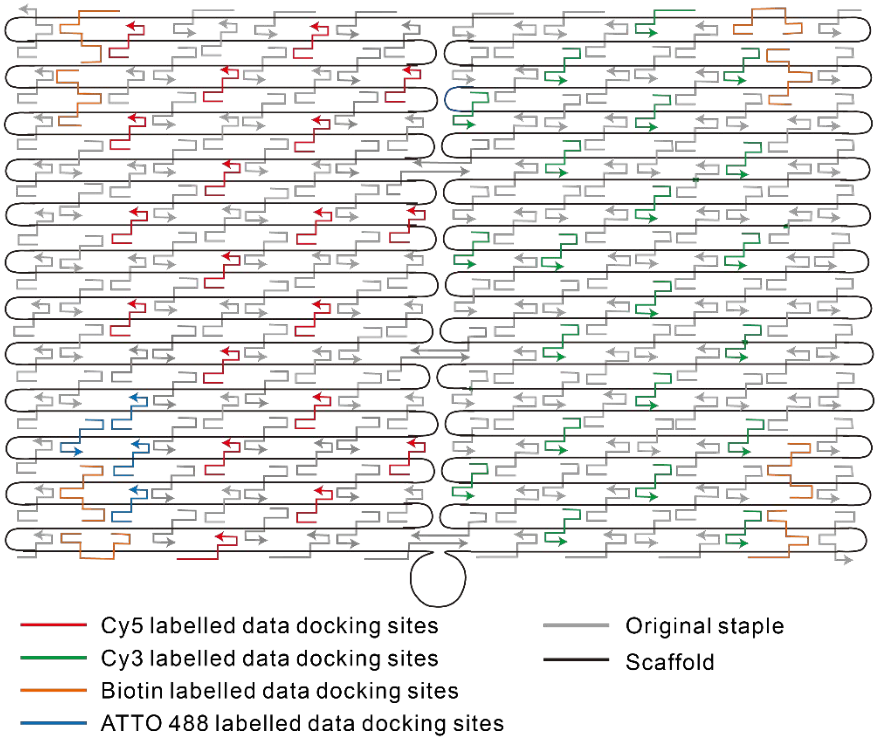


**Figure S2. Detailed design of DNA origami register.**

The red lines represent docking sites of the information strands labeled with Cy5, the green lines represent docking sites of the information strands labeled with Cy3, the orange lines represent docking points of the fixed strands labeled with Biotin, and the blue lines represent docking points of the indicator strands labeled with ATTO 488. The black lines represent the scaffold strand folding mode, and the grey lines represent the staple strands folding mode.

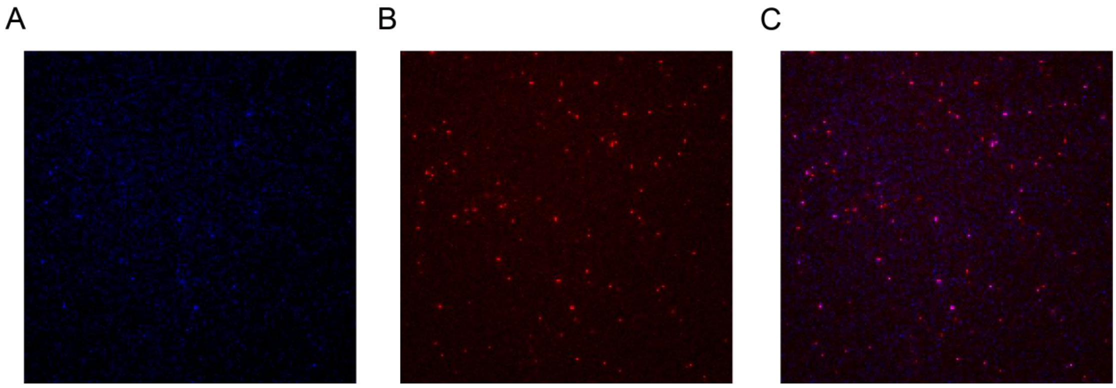


**Figure S3. The fluorescence images showing the colocalization between ATTO 488 and Cy5.**

(**A**) The fluorescence image of the ATTO 488 (blue) showing the position of the DNA origami register.

(**B**) The fluorescence image of Cy5 (Red) showing the position of the data strands.

(**C**) The fluorescence merge image showing the colocalization of the ATTO 488 and Cy5 (magenta).

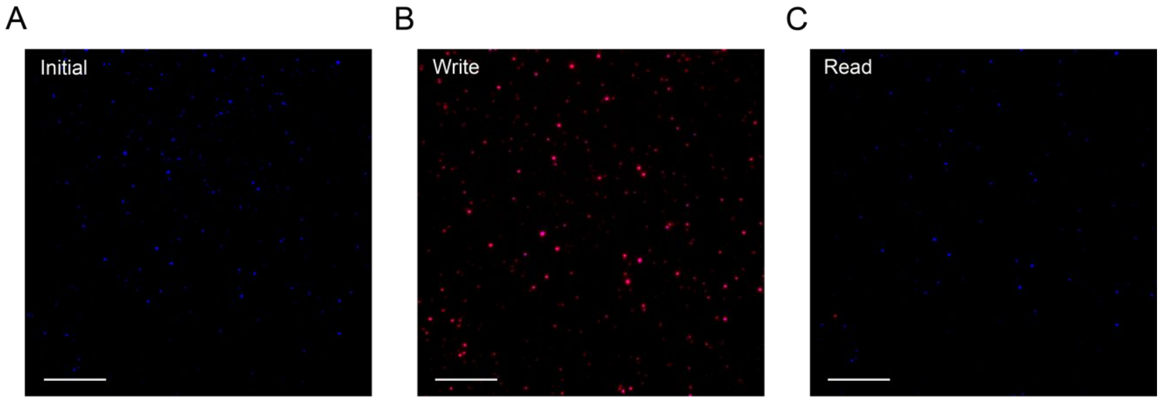


**Figure S4. Single-molecule fluorescence images showing fluorescence changes during the data writing and reading processes.** The blue channel showing the fluorescence ATTO-488 marker and the red channel showing the fluorescence of Cy5 -labeled data strand.

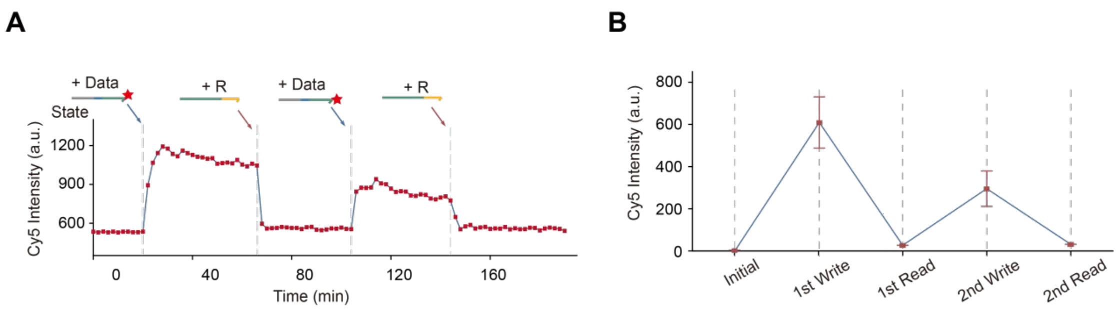


**Figure S5. Representative traces and analysis graphs of repeated writing and reading of data, while $L_{Writing}$ = 5 nt and $L_{Reading}$ = 7 nt.**

(**A**) Representative fluorescence traces showing the dynamic process of repeatedly writing and reading Cy5-labeled data strands on the DNA origami register and the fluorescence changes in the background.

(**B**) Statistical graph of fluorescence intensity of Cy5 at each stage after background subtraction.

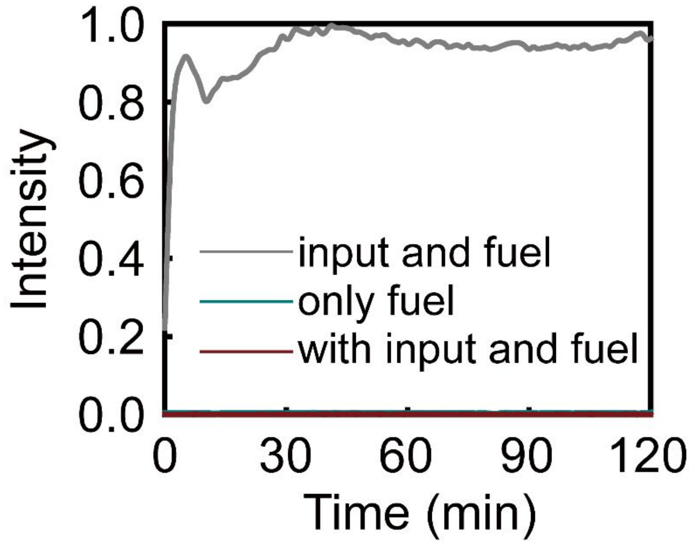


**Figure S6. Amplified dynamic fluorescence signal with the conventional amplifier.**

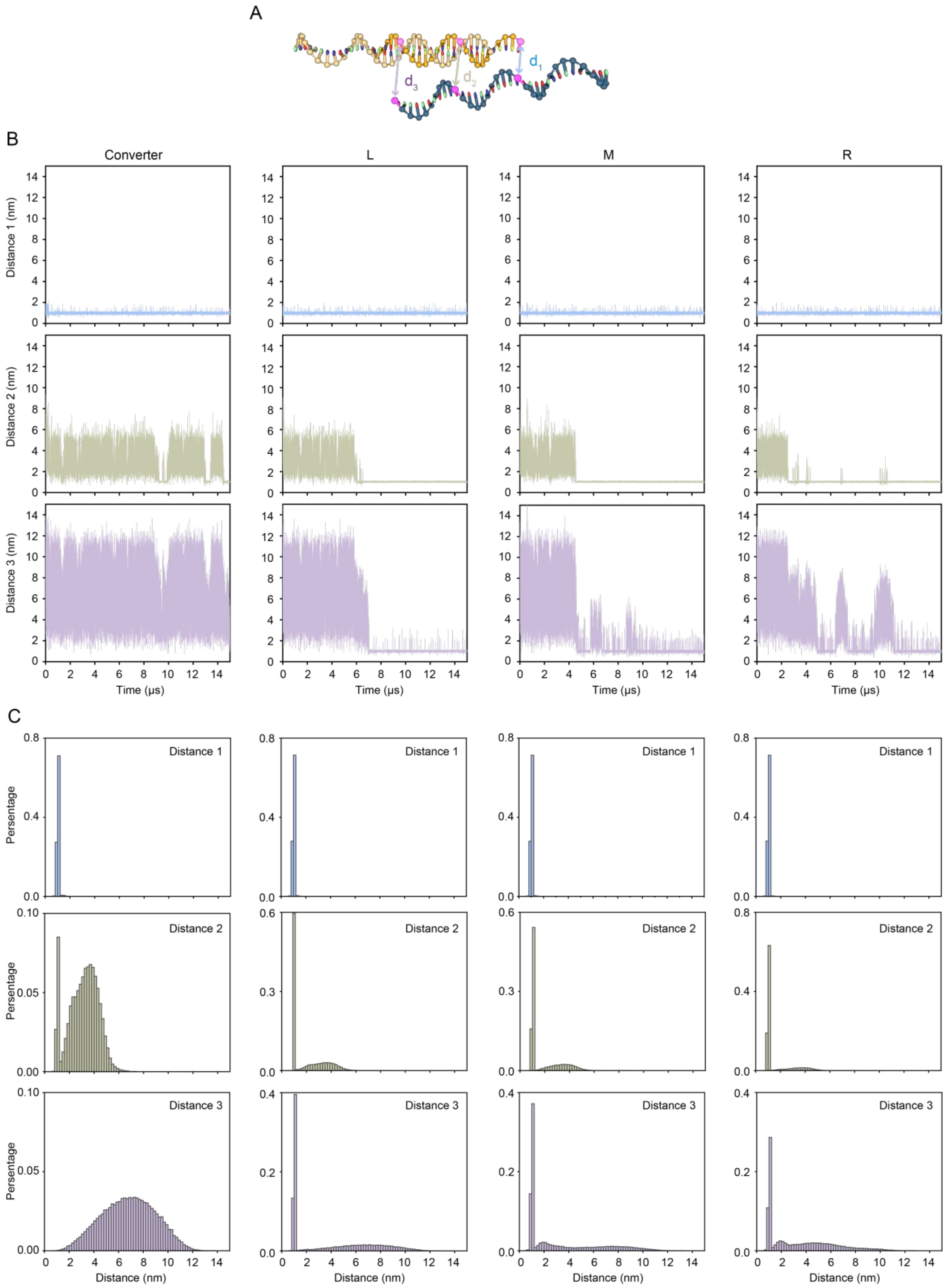


**Figure S7. Dynamic simulation analysis of strand replacement between different converter and input, simulation time trajectory (B) and distance distribution within simulation time (C).**

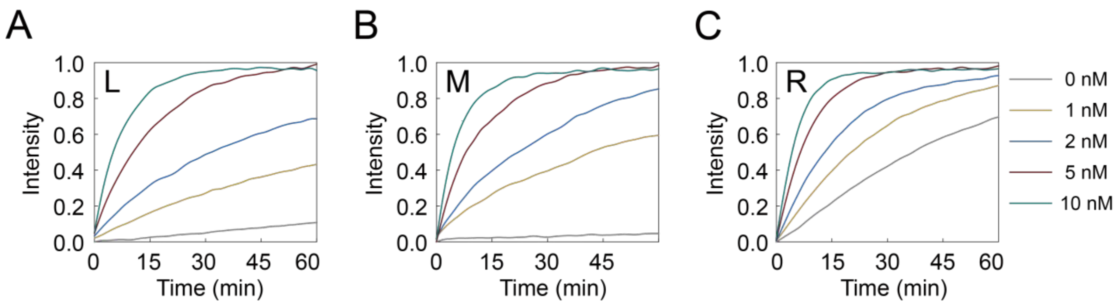


**Figure S8. Dynamic fluorescence signal of the L (A), M (B), and R (C) amplifiers.**

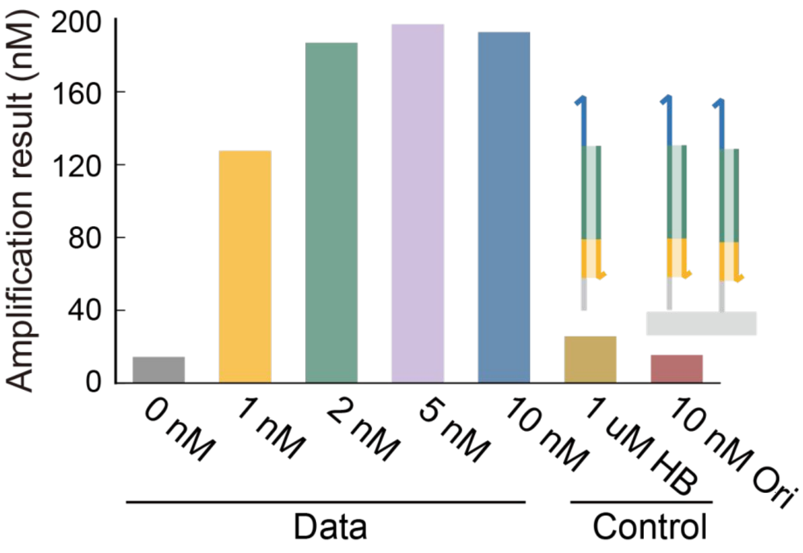


**Figure S9. Amplification results for different concentrations of Data using M converter.** Control experiments involved the introduction of double-stranded DNA extending from the origami memory and the memory itself into the system, assessing whether their inclusion triggers any leakage.

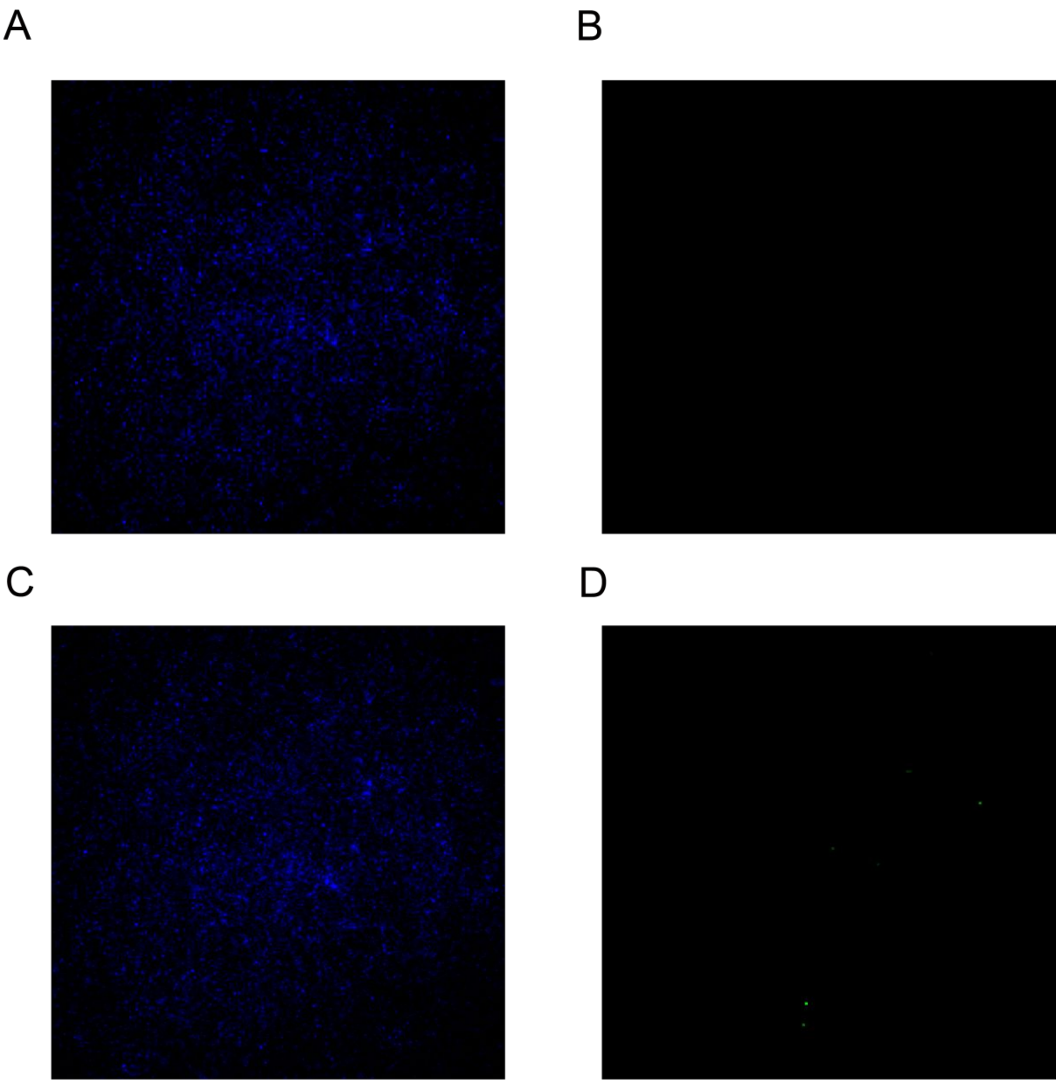


**Figure S10. The fluorescence images showing the fluorescence does not change before and after adding the labelling gate.**

(**A and B**) The fluorescence image of the DNA origami register marker and the Cy3 fluorescence before adding the labelling gate labelled Cy3.

(**C and D**) The fluorescence image of the DNA origami register marker and the Cy3 fluorescence after adding the labelling gate labelled Cy3 (after incubated for 1.5h).

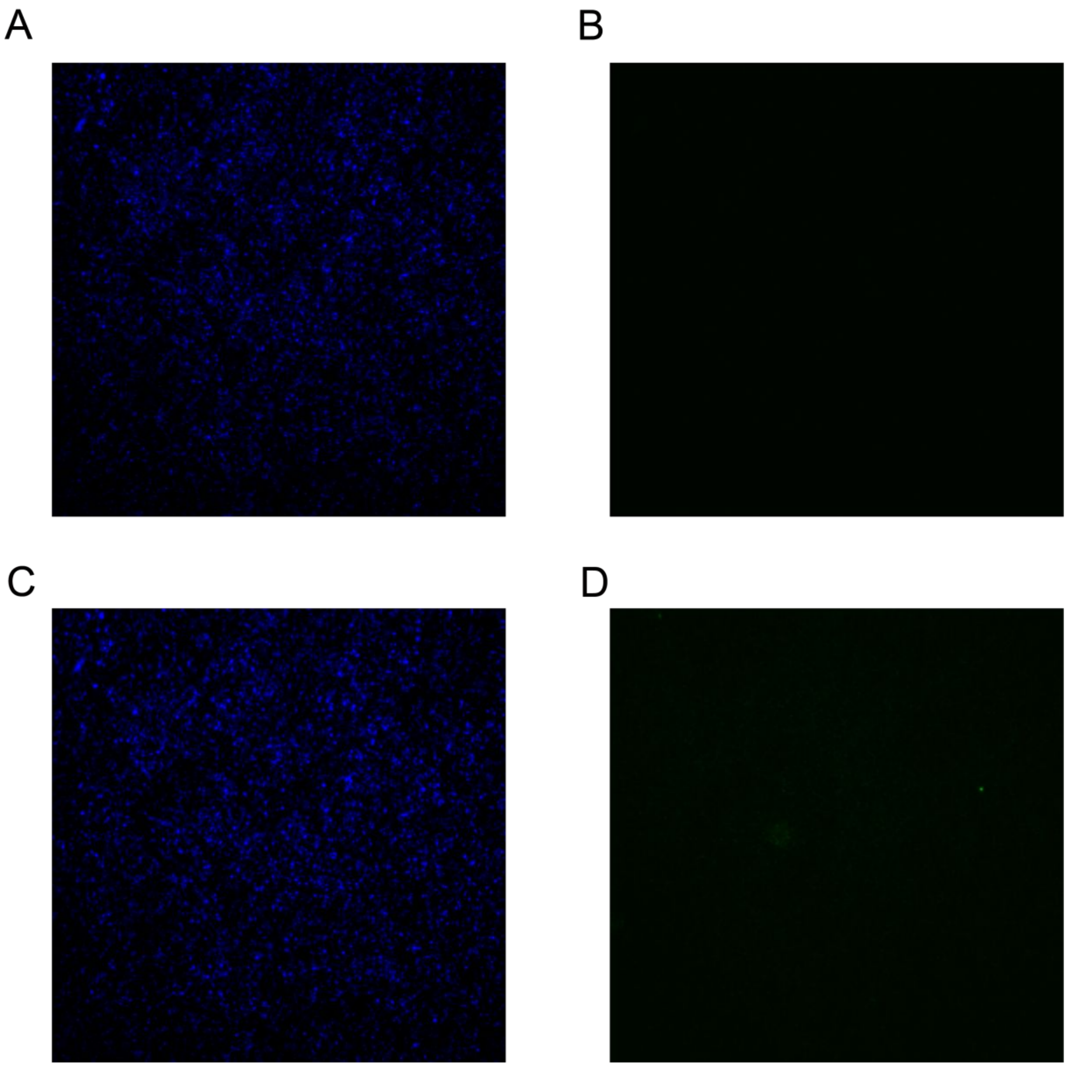


**Figure S11. The fluorescence images showing the fluorescence does not change with all reaction components present except input.**

(**A, B**) The fluorescence image of the DNA origami register marker and the Cy3 fluorescence before adding all reaction components present except input.

(**C, D**) The fluorescence image of the DNA origami register marker and the Cy3 fluorescence after all reaction components present except input (after incubated for 1.5h).

## SUPPORTING TABLES

## Table S1. Sequence of M13mp18 scaffold

| |
|---|
| TTCCCTTCCTTTCTCGCCACGTTCGCCGGCTTTCCCCGTCAAGCTCTAAATCGGGG |
| GCTCCCTTTAGGGTTCCGATTTAGTGCTTTACGGCACCTCGACCCCAAAAAACTTGA |
| TTTGGGTGATGGTTCACGTAGTGGGCCATCGCCCTGATAGACGGTTTTTCGCCCTT |
| TGACGTTGGAGTCCACGTTCTTTAATAGTGGACTCTTGTTCCAAACTGGAACAACAC |
| TCAACCCTATCTCGGGCTATTCTTTTGATTTATAAGGGATTTTGCCGATTTCGGAAC |
| CACCATCAAACAGGATTTTCGCCTGCTGGGGCAAACCAGCGTGGACCGCTTGCTG |
| CAACTCTCTCAGGGCCAGGCGGTGAAGGGCAATCAGCTGTTGCCCGTCTCACTGG |
| TGAAAAGAAAAACCACCCTGGCGCCCAATACGCAAACCGCCTCTCCCCGCGCGTT |
| GGCCGATTCATTAATGCAGCTGGCACGACAGGTTTCCCGACTGGAAAGCGGGCAG |
| TGAGCGCAACGCAATTAATGTGAGTTAGCTCACTCATTAGGCACCCCAGGCTTTAC |
| ACTTTATGCTTCCGGCTCGTATGTTGTGTGGAATTGTGAGCGGATAACAATTTCACA |
| CAGGAAACAGCTATGACCATGATTACGAATTCGAGCTCGGTACCCGGGGATCCTCT |
| AGAGTCGACCTGCAGGCATGCAAGCTTGGCACTGGCCGTCGTTTTACAACGTCGT |
| GACTGGGAAAACCCTGGCGTTACCCAACTTAATCGCCTTGCAGCACATCCCCCTTT |
| CGCCAGCTGGCGTAATAGCGAAGAGGCCCGCACCGATCGCCCTTCCCAACAGTTG |
| CGCAGCCTGAATGGCGAATGGCGCTTTGCCTGGTTTCCGGCACCAGAAGCGGTGC |
| CGGAAAGCTGGCTGGAGTGCGATCTTCCTGAGGCCGATACTGTCGTCGTCCCCTC |
| AAACTGGCAGATGCACGGTTACGATGCGCCCATCTACACCAACGTGACCTATCCCA |
| TTACGGTCAATCCGCCGTTTGTTCCCACGGAGAATCCGACGGGTTGTTACTCGCTC |
| ACATTTAATGTTGATGAAAGCTGGCTACAGGAAGGCCAGACGCGAATTATTTTTGAT |
| GGCGTTCCTATTGGTTAAAAAATGAGCTGATTTAACAAAAATTTAATGCGAATTTTAA |
| CAAAATATTAACGTTTACAATTTAAATATTTGCTTATACAATCTTCCTGTTTTTGGGGC |
| TTTTCTGATTATCAACCGGGGTACATATGATTGACATGCTAGTTTTACGATTACCGTT |
| CATCGATTCTCTTGTTTGCTCCAGACTCTCAGGCAATGACCTGATAGCCTTTGTAGA |
| TCTCTCAAAAATAGCTACCCTCTCCGGCATTAATTTATCAGCTAGAACGGTTGAATA |
| TCATATTGATGGTGATTTGACTGTCTCCGGCCTTTCTCACCCTTTTGAATCTTTACCT |
| ACACATTACTCAGGCATTGCATTTAAAATATATGAGGGTTCTAAAAATTTTTATCCTT |
| GCGTTGAAATAAAGGCTTCTCCCGCAAAAGTATTACAGGGTCATAATGTTTTTGGTA |
| CAACCGATTTAGCTTTATGCTCTGAGGCTTTATTGCTTAATTTTGCTAATTCTTTGCC |
| TTGCCTGTATGATTTATTGGATGTTAATGCTACTACTATTAGTAGAATTGATGCCACC |
| TTTTCAGCTCGCGCCCCAAATGAAAATATAGCTAAACAGGTTATTGACCATTTGCGA |
| AATGTATCTAATGGTCAAACTAAATCTACTCGTTCGCAGAATTGGGAATCAACTGTT |
| ATATGGAATGAAACTTCCAGACACCGTACTTTAGTTGCATATTTAAAACATGTTGAG |
| CTACAGCATTATATTCAGCAATTAAGCTCTAAGCCATCCGCAAAAATGACCTCTTAT |

CAAAAGGAGCAATTAAAGGTACTCTCTAATCCTGACCTGTTGGAGTTTGCTTCCGGT
CTGGTTCGCTTTGAAGCTCGAATTAAAACGCGATATTTGAAGTCTTTCGGGCTTCCT
CTTAATCTTTTTGATGCAATCCGCTTTGCTTCTGACTATAATAGTCAGGGTAAAGACC
TGATTTTTGATTTATGGTCATTCTCGTTTTCTGAACTGTTTAAAGCATTTGAGGGGGA
TTCAATGAATATTTATGACGATTCCGCAGTATTGGACGCTATCCAGTCTAAACATTTT
ACTATTACCCCCTCTGGCAAAACTTCTTTTGCAAAAGCCTCTCGCTATTTTGGTTTTT
ATCGTCGTCTGGTAAACGAGGGTTATGATAGTGTTGCTCTTACTATGCCTCGTAATT
CCTTTTGGCGTTATGTATCTGCATTAGTTGAATGTGGTATTCCTAAATCTCAACTGAT
GAATCTTTCTACCTGTAATAATGTTGTTCCGTTAGTTCGTTTTATTAACGTAGATTTTT
CTTCCCAACGTCCTGACTGGTATAATGAGCCAGTTCTTAAAATCGCATAAGGTAATT
CACAATGATTAAAGTTGAAATTAAACCATCTCAAGCCCAATTTACTACTCGTTCTGGT
GTTTCTCGTCAGGGCAAGCCTTATTCACTGAATGAGCAGCTTTGTTACGTTGATTTG
GGTAATGAATATCCGGTTCTTGTCAAGATTACTCTTGATGAAGGTCAGCCAGCCTAT
GCGCCTGGTCTGTACACCGTTCATCTGTCCTCTTTCAAAGTTGGTCAGTTCGGTTCC
CTTATGATTGACCGTCTGCGCCTCGTTCCGGCTAAGTAACATGGAGCAGGTCGCGG
ATTTCGACACAATTTATCAGGCGATGATACAAATCTCCGTTGTACTTTGTTTCGCGC
TTGGTATAATCGCTGGGGGTCAAAGATGAGTGTTTTAGTGTATTCTTTTGCCTCTTT
CGTTTTAGGTTGGTGCCTTCGTAGTGGCATTACGTATTTTACCCGTTTAATGGAAAC
TTCCTCATGAAAAAGTCTTTAGTCCTCAAAGCCTCTGTAGCCGTTGCTACCCTCGTT
CCGATGCTGTCTTTCGCTGCTGAGGGTGACGATCCCGCAAAAGCGGCCTTTAACTC
CCTGCAAGCCTCAGCGACCGAATATATCGGTTATGCGTGGGCGATGGTTGTTGTCA
TTGTCGGCGCAACTATCGGTATCAAGCTGTTTAAGAAATTCACCTCGAAAGCAAGCT
GATAAACCGATACAATTAAAGGCTCCTTTTGGAGCCTTTTTTTTGGAGATTTTCAACG
TGAAAAAATTATTATTCGCAATTCCTTTAGTTGTTCCTTTCTATTCTCACTCCGCTGA
AACTGTTGAAAGTTGTTTAGCAAAATCCCATACAGAAAATTCATTTACTAACGTCTGG
AAAGACGACAAAACTTTAGATCGTTACGCTAACTATGAGGGCTGTCTGTGGAATGCT
ACAGGCGTTGTAGTTTGTACTGGTGACGAAACTCAGTGTTACGGTACATGGGTTCC
TATTGGGCTTGCTATCCCTGAAAATGAGGGTGGTGGCTCTGAGGGTGGCGGTTCT
GAGGGTGGCGGTTCTGAGGGTGGCGGTACTAAACCTCCTGAGTACGGTGATACAC
CTATTCCGGGCTATACTTATATCAACCCTCTCGACGGCACTTATCCGCCTGGTACTG
AGCAAAACCCCGCTAATCCTAATCCTTCTCTTGAGGAGTCTCAGCCTCTTAATACTT
TCATGTTTCAGAATAATAGGTTCCGAAATAGGCAGGGGGCATTAACTGTTTATACGG
GCACTGTTACTCAAGGCACTGACCCCGTTAAAACTTATTACCAGTACACTCCTGTAT
CATCAAAAGCCATGTATGACGCTTACTGGAACGGTAAATTCAGAGACTGCGCTTTC
CATTCTGGCTTTAATGAGGATTTATTTGTTTGTGAATATCAAGGCCAATCGTCTGAC
CTGCCTCAACCTCCTGTCAATGCTGGCGGCGGCTCTGGTGGTGGTTCTGGTGGCG
GCTCTGAGGGTGGTGGCTCTGAGGGTGGCGGTTCTGAGGGTGGCGGCTCTGAGG
GAGGCGGTTCCGGTGGTGGCTCTGGTTCCGGTGATTTTGATTATGAAAAGATGGCA

AACGCTAATAAGGGGGCTATGACCGAAAATGCCGATGAAAACGCGCTACAGTCTGA
CGCTAAAGGCAAACTTGATTCTGTCGCTACTGATTACGGTGCTGCTATCGATGGTTT
CATTGGTGACGTTTCCGGCCTTGCTAATGGTAATGGTGCTACTGGTGATTTTGCTG
GCTCTAATTCCCAAATGGCTCAAGTCGGTGACGGTGATAATTCACCTTTAATGAATA
ATTTCCGTCAATATTTACCTTCCCTCCCTCAATCGGTTGAATGTCGCCCTTTTGTCTT
TGGCGCTGGTAAACCATATGAATTTTCTATTGATTGTGACAAAATAAACTTATTCCGT
GGTGTCTTTGCGTTTCTTTTATATGTTGCCACCTTTATGTATGTATTTTCTACGTTTG
CTAACATACTGCGTAATAAGGAGTCTTAATCATGCCAGTTCTTTTGGGTATTCCGTT
ATTATTGCGTTTCCTCGGTTTCCTTCTGGTAACTTTGTTCGGCTATCTGCTTACTTTT
CTTAAAAAGGGCTTCGGTAAGATAGCTATTGCTATTTCATTGTTTCTTGCTCTTATTA
TTGGGCTTAACTCAATTCTTGTGGGTTATCTCTCTGATATTAGCGCTCAATTACCCTC
TGACTTTGTTCAGGGTGTTCAGTTAATTCTCCCGTCTAATGCGCTTCCCTGTTTTTAT
GTTATTCTCTCTGTAAAGGCTGCTATTTTCATTTTTGACGTTAAACAAAAAATCGTTT
CTTATTTGGATTGGGATAAATAATATGGCTGTTTATTTTGTAACTGGCAAATTAGGCT
CTGGAAAGACGCTCGTTAGCGTTGGTAAGATTCAGGATAAAATTGTAGCTGGGTGC
AAAATAGCAACTAATCTTGATTTAAGGCTTCAAAACCTCCCGCAAGTCGGGAGGTTC
GCTAAAACGCCTCGCGTTCTTAGAATACCGGATAAGCCTTCTATATCTGATTTGCTT
GCTATTGGGCGCGGTAATGATTCCTACGATGAAAATAAAAACGGCTTGCTTGTTCTC
GATGAGTGCGGTACTTGGTTTAATACCCGTTCTTGGAATGATAAGGAAAGACAGCC
GATTATTGATTGGTTTCTACATGCTCGTAAATTAGGATGGGATATTATTTTTCTTGTT
CAGGACTTATCTATTGTTGATAAACAGGCGCGTTCTGCATTAGCTGAACATGTTGTT
TATTGTCGTCGTCTGGACAGAATTACTTTACCTTTTGTCGGTACTTTATATTCTCTTA
TTACTGGCTCGAAAATGCCTCTGCCTAAATTACATGTTGGCGTTGTTAAATATGGCG
ATTCTCAATTAAGCCCTACTGTTGAGCGTTGGCTTTATACTGGTAAGAATTTGTATAA
CGCATATGATACTAAACAGGCTTTTTCTAGTAATTATGATTCCGGTGTTTATTCTTAT
TTAACGCCTTATTTATCACACGGTCGGTATTTCAAACCATTAAATTTAGGTCAGAAGA
TGAAATTAACTAAAATATATTTGAAAAAGTTTTCTCGCGTTCTTTGTCTTGCGATTGG
ATTTGCATCAGCATTTACATATAGTTATATAACCCAACCTAAGCCGGAGGTTAAAAA
GGTAGTCTCTCAGACCTATGATTTTGATAAATTCACTATTGACTCTTCTCAGCGTCTT
AATCTAAGCTATCGCTATGTTTTCAAGGATTCTAAGGGAAAATTAATTAATAGCGAC
GATTTACAGAAGCAAGGTTATTCACTCACATATATTGATTTATGTACTGTTTCCATTA
AAAAAGGTAATTCAAATGAAATTGTTAAATGTAATTAATTTTGTTTTCTTGATGTTTGT
TTCATCATCTTCTTTTGCTCAGGTAATTGAAATGAATAATTCGCCTCTGCGCGATTTT
GTAACTTGGTATTCAAAGCAATCAGGCGAATCCGTTATTGTTTCTCCCGATGTAAAA
GGTACTGTTACTGTATATTCATCTGACGTTAAACCTGAAAATCTACGCAATTTCTTTA
TTTCTGTTTTACGTGCAAATAATTTTGATATGGTAGGTTCTAACCCTTCCATTATTCA
GAAGTATAATCCAAACAATCAGGATTATATTGATGAATTGCCATCATCTGATAATCAG
GAATATGATGATAATTCCGCTCCTTCTGGTGGTTTCTTTGTTCCGCAAAATGATAAT

GTTACTCAAACTTTTAAAATTAATAACGTTCGGGCAAAGGATTTAATACGAGTTGTCG
AATTGTTTGTAAAGTCTAATACTTCTAAATCCTCAAATGTATTATCTATTGACGGCTC
TAATCTATTAGTTGTTAGTGCTCCTAAAGATATTTTAGATAACCTTCCTCAATTCCTTT
CAACTGTTGATTTGCCAACTGACCAGATATTGATTGAGGGTTTGATATTTGAGGTTC
AGCAAGGTGATGCTTTAGATTTTTCATTTGCTGCTGGCTCTCAGCGTGGCACTGTTG
CAGGCGGTGTTAATACTGACCGCCTCACCTCTGTTTTATCTTCTGCTGGTGGTTCGT
TCGGTATTTTTAATGGCGATGTTTTAGGGCTATCAGTTCGCGCATTAAAGACTAATA
GCCATTCAAAAATATTGTCTGTGCCACGTATTCTTACGCTTTCAGGTCAGAAGGGTT
CTATCTCTGTTGGCCAGAATGTCCCTTTTATTACTGGTCGTGTGACTGGTGAATCTG
CCAATGTAAATAATCCATTTCAGACGATTGAGCGTCAAAATGTAGGTATTTCCATGA
GCGTTTTTCCTGTTGCAATGGCTGGCGGTAATATTGTTCTGGATATTACCAGCAAGG
CCGATAGTTTG

## Table S2. Sequences of DNA origami register staples

| | |
|---|---|
| 1 | CAAGCCCAATAGGAACCCATGTACAAACAGTT |
| 2 | AATGCCCCGTAACAGTGCCCGTATCTCCCTCA |
| 3 | TGCCTTGACTGCCTATTTCGGAACAGGGATAG |
| 5 | AACCAGAGACCCTCAGAACCGCCAGGGGTCAG |
| 6 | TTATTCATAGGGAAGGTAAATATTCATTCAGT |
| 7 | CATAACCCGAGGCATAGTAAGAGCTTTTTAAG |
| 8 | ATTGAGGGTAAAGGTGAATTATCAATCACCGG |
| 9 | AAAAGTAATATCTTACCGAAGCCCTTCCAGAG |
| 10 | GCAATAGCGCAGATAGCCGAACAATTCAACCG |
| 11 | CCTAATTTACGCTAACGAGCGTCTAATCAATA |
| 12 | TCTTACCAGCCAGTTACAAAATAAATGAAATA |
| 14 | CTAATTTATCTTTCCTTATCATTCATCCTGAA |
| 15 | GCGTTATAGAAAAAGCCTGTTTAGAAGGCCGG |
| 16 | GCTCATTTTCGCATTAAATTTTTGAGCTTAGA |
| 17 | AATTACTACAAATTCTTACCAGTAATCCCATC |
| 18 | TTAAGACGTTGAAAACATAGCGATAACAGTAC |
| 19 | TAGAATCCCTGAGAAGAGTCAATAGGAATCAT |
| 20 | CTTTTACACAGATGAATATACAGTAAACAATT |
| 21 | TTTAACGTTCGGGAGAAACAATAATTTTCCCT |
| 23 | GGATTTAGCGTATTAAATCCTTTGTTTTCAGG |
| 24 | ACGAACCAAAACATCGCCATTAAATGGTGGTT |
| 25 | GAACGTGGCGAGAAAGGAAGGGAACAAACTAT |
| 26 | TAGCCCTACCAGCAGAAGATAAAAACATTTGA |
| 27 | CGGCCTTGCTGGTAATATCCAGAACGAACTGA |
| 28 | CTCAGAGCCACCACCCTCATTTTCCTATTATT |
| 30 | AGTGTACTTGAAAGTATTAAGAGGCCGCCACC |
| 31 | GCCACCACTCTTTTCATAATCAAACCGTCACC |
| 32 | GTTTGCCACCTCAGAGCCGCCACCGATACAGG |
| 34 | AGCGCCAACCATTTGGGAATTAGATTATTAGC |
| 35 | GAAGGAAAATAAGAGCAAGAAACAACAGCCAT |
| 36 | GCCCAATACCGAGGAAACGCAATAGGTTTACC |
| 38 | TATTTTGCTCCCAATCCAAATAAGTGAGTTAA |
| 39 | GGTATTAAGAACAAGAAAAATAATTAAAGCCA |
| 40 | TAAGTCCTACCAAGTACCGCACTCTTAGTTGC |
| 42 | AGGCGTTACAGTAGGGCTTAATTGACAATAGA |
| 43 | ATCAAAATCGTCGCTATTAATTAACGGATTCG |
| 44 | CTGTAAATCATAGGTCTGAGAGACGATAAATA |
| 46 | ACAGAAATCTTTGAATACCAAGTTCCTTGCTT |
| 47 | TTATTAATGCCGTCAATAGATAATCAGAGGTG |
| 48 | AGATTAGATTTAAAAGTTTGAGTACACGTAAA |
| 50 | GAATGGCTAGTATTAACACCGCCTCAACTAAT |

| | |
|---|---|
| 51 | CCGCCAGCCATTGCAACAGGAAAAATATTTTT |
| 53 | CCTCAAGAATACATGGCTTTTGATAGAACCAC |
| 54 | TAAGCGTCGAAGGATTAGGATTAGTACCGCCA |
| 56 | TCGGCATTCCGCCGCCAGCATTGACGTTCCAG |
| 57 | AATCACCAAATAGAAAATTCATATATAACGGA |
| 58 | TCACAATCGTAGCACCATTACCATCGTTTTCA |
| 60 | ATCAGAGAAAGAACTGGCATGATTTTATTTTG |
| 61 | TTTTGTTTAAGCCTTAAATCAAGAATCGAGAA |
| 62 | AGGTTTTGAACGTCAAAAATGAAAGCGCTAAT |
| 64 | AATGCAGACCGTTTTTATTTTCATCTTGCGGG |
| 65 | CATATTTAGAAATACCGACCGTGTTACCTTTT |
| 66 | AATGGTTTACAACGCCAACATGTAGTTCAGCT |
| 68 | AAATCAATGGCTTAGGTTGGGTTACTAAATTT |
| 69 | GCGCAGAGATATCAAAATTATTTGACATTATC |
| 70 | AACCTACCGCGAATTATTCATTTCCAGTACAT |
| 72 | CTAAAATAGAACAAAGAAACCACCAGGGTTAG |
| 73 | GCCACGCTATACGTGGCACAGACAACGCTCAT |
| 74 | GCGTAAGAGAGAGCCAGCAGCAAAAAGGTTAT |
| 75 | GGAAATACCTACATTTTGACGCTCACCTGAAA |
| 79 | TGAGGCAGGCGTCAGACTGTAGCGTAGCAAGG |
| 82 | ACGCAAAGGTCACCAATGAAACCAATCAAGTT |
| 83 | TTATTACGGTCAGAGGGTAATTGAATAGCAGC |
| 84 | TGAACAAACAGTATGTTAGCAAACTAAAAGAA |
| 86 | GAGGCGTTAGAGAATAACATAAAAGAACACCC |
| 87 | TCATTACCCGACAATAAACAACATATTTAGGC |
| 88 | CCAGACGAGCGCCCAATAGCAAGCAAGAACGC |
| 90 | TTTTAGTTTTTCGAGCCAGTAATAAATTCTGT |
| 91 | TATGTAAACCTTTTTTAATGGAAAAATTACCT |
| 92 | TTGAATTATGCTGATGCAAATCCACAAATATA |
| 94 | TGGATTATGAAGATGATGAAACAAAATTTCAT |
| 95 | CGGAATTATTGAAAGGAATTGAGGTGAAAAAT |
| 112 | CCGAAATCCGAAAATCCTGTTTGAAGCCGGAA |
| 113 | CCAGCAGGGGCAAAATCCCTTATAAAGCCGGC |
| 115 | GCTCACAATGTAAAGCCTGGGGTGGGTTTGCC |
| 116 | TTCGCCATTGCCGGAAACCAGGCATTAAATCA |
| 117 | GCTTCTGGTCAGGCTGCGCAACTGTGTTATCC |
| 118 | GTTAAAATTTTAACCAATAGGAACCCGGCACC |
| 119 | AGACAGTCATTCAAAAGGGTGAGAAGCTATAT |
| 120 | AGGTAAAGAAATCACCATCAATATAATATTTT |
| 121 | TTTCATTTGGTCAATAACCTGTTTATATCGCG |
| 122 | TCGCAAATGGGGCGCGAGCTGAAATAATGTGT |
| 124 | AAGAGGAACGAGCTTCAAAGCGAAGATACATT |
| 125 | GGAATTACTCGTTTACCAGACGACAAAAGATT |

| | |
|---|---|
| 126 | GAATAAGGACGTAACAAAGCTGCTCTAAAACA |
| 127 | CCAAATCACTTGCCCTGACGAGAACGCCAAAA |
| 129 | AAACGAAATGACCCCCAGCGATTATTCATTAC |
| 130 | CTTAAACATCAGCTTGCTTTCGAGCGTAACAC |
| 131 | TCGGTTTAGCTTGATACCGATAGTCCAACCTA |
| 132 | TGAGTTTCGTCACCAGTACAAACTTAATTGTA |
| 133 | CCCCGATTTAGAGCTTGACGGGGAAATCAAAA |
| 135 | GAGTTGCACGAGATAGGGTTGAGTAAGGGAGC |
| 136 | GTGAGCTAGTTTCCTGTGTGAAATTTGGGAAG |
| 137 | TCATAGCTACTCACATTAATTGCGCCCTGAGA |
| 139 | GAAGATCGGTGCGGGCCTCTTCGCAATCATGG |
| 140 | AAATAATTTTAAATTGTAAACGTTGATATTCA |
| 141 | GCAAATATCGCGTCTGGCCTTCCTGGCCTCAG |
| 143 | TATATTTTAGCTGATAAATTAATGTTGTATAA |
| 144 | TCAATTCTTTTAGTTTGACCATTACCAGACCG |
| 145 | CGAGTAGAACTAATAGTAGTAGCAAACCCTCA |
| 147 | TCAGAAGCCTCCAACAGGTCAGGATCTGCGAA |
| 148 | CCAAAATATAATGCAGATACATAAACACCAGA |
| 149 | CATTCAACGCGAGAGGCTTTTGCATATTATAG |
| 151 | AGTAATCTTAAATTGGGCTTGAGAGAATACCA |
| 152 | GCGAAACATGCCACTACGAAGGCATGCGCCGA |
| 153 | ATACGTAAAAGTACAACGGAGATTTCATCAAG |
| 155 | AAAAAAGGACAACCATCGCCCACGCGGGTAAA |
| 156 | TGTAGCATTCCACAGACAGCCCTCATCTCCAA |
| 158 | AGTTTGGAGCCCTTCACCGCCTGGTTGCGCTC |
| 159 | AGCTGATTACAAGAGTCCACTATTGAGGTGCC |
| 161 | CCCGGGTACTTTCCAGTCGGGAAACGGGCAAC |
| 162 | CAGCTGGCGGACGACGACAGTATCGTAGCCAG |
| 163 | GTTTGAGGGAAAGGGGGATGTGCTAGAGGATC |
| 165 | AGAAAAGCAACATTAAATGTGAGCATCTGCCA |
| 166 | GGTAGCTAGGATAAAAATTTTTAGTTAACATC |
| 167 | CAACGCAATTTTTGAGAGATCTACTGATAATC |
| 169 | TCCATATACATACAGGCAAGGCAACTTTATTT |
| 170 | TACCTTTAAGGTCTTTACCCTGACAAAGAAGT |
| 171 | CAAAAATCATTGCTCCTTTTGATAAGTTTCAT |
| 173 | AAAGATTCAGGGGGTAATAGTAAACCATAAAT |
| 174 | TTTCAACTATAGGCTGGCTGACCTTGTATCAT |
| 175 | CCAGGCGCTTAATCATTGTGAATTACAGGTAG |
| 177 | TTTCATGAAAATTGTGTCGAAATCTGTACAGA |
| 178 | ATATATTCTTTTTTCACGTTGAAAATAGTTAG |
| 179 | AATAATAAGGTCGCTGAGGCTTGCAAAGACTT |
| 180 | CGTAACGATCTAAAGTTTTGTCGTGAATTGCG |
| 188 | ACCCGTCGTCATATGTACCCCGGTAAAGGCTA |

| | |
|---|---|
| 189 | CATGTCAAGATTCTCCGTGGGAACCGTTGGTG |
| 191 | CTGTAATATTGCCTGAGAGTCTGGAAAACTAG |
| 192 | CAAAATTAAAGTACGGTGTCTGGAAGAGGTCA |
| 193 | TGCAACTAAGCAATAAAGCCTCAGTTATGACC |
| 195 | AAACAGTTGATGGCTTAGAGCTTATTTAAATA |
| 196 | ACTGGATAACGGAACAACATTATTACCTTATG |
| 197 | ACGAACTAGCGTCCAATACTGCGGAATGCTTT |
| 199 | CTTTGAAAAGAACTGGCTCATTATTTAATAAA |
| 200 | GCTCCATGAGAGGCTTTGAGGACTAGGGAGTT |

**Supplementary Table S3. Sequences of staples at complementary to Cy5-data strands**

| | |
|---|---|
| 114 | GATGAGATGTATATGAGAGAGGAGTAGAAGTTTTTGCATAAAGTTCCACACAACATACGAAGCGCCA |
| 123 | GATGAGATGTATATGAGAGAGGAGTAGAAGTTTTTTTTTAATTGCCCGAAAGACTTCAAAACACTAT |
| 128 | GATGAGATGTATATGAGAGAGGAGTAGAAGTTTTTCTCATCTTGAGGCAAAAGAATACAGTGAATTT |
| 134 | GATGAGATGTATATGAGAGAGGAGTAGAAGTTTTTGAATAGCCGCAAGCGGTCCACGCTCCTAATGA |
| 138 | GATGAGATGTATATGAGAGAGGAGTAGAAGTTTTTGGCGATCGCACTCCAGCCAGCTTTGCCATCAA |
| 142 | GATGAGATGTATATGAGAGAGGAGTAGAAGTTTTTACCGTTCTAAATGCAATGCCTGAGAGGTGGCA |
| 146 | GATGAGATGTATATGAGAGAGGAGTAGAAGTTTTTGAAGCAAAAAAGCGGATTGCATCAGATAAAAA |
| 150 | GATGAGATGTATATGAGAGAGGAGTAGAAGTTTTTACGAGTAGTGACAAGAACCGGATATACCAAGC |
| 154 | GATGAGATGTATATGAGAGAGGAGTAGAAGTTTTTCAATGACACTCCAAAAGGAGCCTTACAACGCC |
| 157 | GATGAGATGTATATGAGAGAGGAGTAGAAGTTTTTGTAAAGCACTAAATCGGAACCCTAGTTGTTCC |
| 160 | GATGAGATGTATATGAGAGAGGAGTAGAAGTTTTTACTGCCCGCCGAGCTCGAATTCGTTATTACGC |
| 164 | GATGAGATGTATATGAGAGAGGAGTAGAAGTTTTTCTTTCATCCCCAAAAACAGGAAGACCGGAGAG |
| 168 | GATGAGATGTATATGAGAGAGGAGTAGAAGTTTTTCAATAAATACAGTTGATTCCCAATTTAGAGAG |

| | |
|---|---|
| 172 | GATGAGATGTATATGAGAGAGGAGTAGAAGTTTTTTTTGCCAGATCAGTTGAG<br>ATTTAGTGGTTTAA |
| 176 | GATGAGATGTATATGAGAGAGGAGTAGAAGTTTTTCGCCTGATGGAAGTTTCC<br>ATTAAACATAACCG |
| 182 | GATGAGATGTATATGAGAGAGGAGTAGAAGTTTTTTGGACTCCCTTTTCACCA<br>GTGAGACCTGTCGT |
| 186 | GATGAGATGTATATGAGAGAGGAGTAGAAGTTTTTATTAAGTTCGCATCGTAAC<br>CGTGCGAGTAACA |
| 190 | GATGAGATGTATATGAGAGAGGAGTAGAAGTTTTTTCAGGTCACTTTTGCGGG<br>AGAAGCAGAATTAG |
| 194 | GATGAGATGTATATGAGAGAGGAGTAGAAGTTTTTTTTTTGCGCAGAAAACGA<br>GAATGAATGTTTAG |
| 198 | GATGAGATGTATATGAGAGAGGAGTAGAAGTTTTTCGATTTTAGAGGACAGAT<br>GAACGGCGCGACCT |
| 202 | GATGAGATGTATATGAGAGAGGAGTAGAAGTTTTTAAAGGCCGAAAGGAACAA<br>CTAAAGCTTTCCAG |

**Supplementary Table S4. Sequences of staples at complementary to Cy3-data strands**

| | |
|---|---|
| 4 | TGGTATGAGGAAGATAGTGGAGTAGAAGTTTTTGAGCCGCCCCACCACCGGAACCGCGACGGAAA |
| 13 | TGGTATGAGGAAGATAGTGGAGTAGAAGTTTTTATCGGCTGCGAGCATGTAGAAACCTATCATAT |
| 22 | TGGTATGAGGAAGATAGTGGAGTAGAAGTTTTTCGACAACTAAGTATTAGACTTTACAATACCGA |
| 29 | TGGTATGAGGAAGATAGTGGAGTAGAAGTTTTTCTGAAACAGGTAATAAGTTTTAACCCCTCAGA |
| 33 | TGGTATGAGGAAGATAGTGGAGTAGAAGTTTTTGACTTGAGAGACAAAAGGGCGACAAGTTACCA |
| 37 | TGGTATGAGGAAGATAGTGGAGTAGAAGTTTTTATTATTTAACCCAGCTACAATTTTCAAGAACG |
| 41 | TGGTATGAGGAAGATAGTGGAGTAGAAGTTTTTACGCTCAAAATAAGAATAAACACCGTGAATTT |
| 45 | TGGTATGAGGAAGATAGTGGAGTAGAAGTTTTTCCTGATTGAAAGAAATTGCGTAGACCCGAACG |
| 49 | TGGTATGAGGAAGATAGTGGAGTAGAAGTTTTTAGGCGGTCATTAGTCTTTAATGCGCAATATTA |
| 52 | TGGTATGAGGAAGATAGTGGAGTAGAAGTTTTTCCCTCAGAACCGCCACCCTCAGAACTGAGACT |
| 55 | TGGTATGAGGAAGATAGTGGAGTAGAAGTTTTTCACCAGAGTTCGGTCATAGCCCCCGCCAGCAA |
| 59 | TGGTATGAGGAAGATAGTGGAGTAGAAGTTTTTATACCCAAGATAACCCACAAGAATAAACGATT |
| 63 | TGGTATGAGGAAGATAGTGGAGTAGAAGTTTTTCAAGCAAGACGCGCCTGTTTATCAAGAATCGC |

| | |
|---|---|
| 67 | TGGTATGAGGAAGATAGTGGAGTAGAAGTTTTTTAACCTCCATATGTGAGTGAATAAACAAAATC |
| 71 | TGGTATGAGGAAGATAGTGGAGTAGAAGTTTTTATTTTGCGTCTTTAGGAGCACTAAGCAACAGT |
| 77 | TGGTATGAGGAAGATAGTGGAGTAGAAGTTTTTTGCTCAGTCAGTCTCTGAATTTACCAGGAGGT |
| 81 | TGGTATGAGGAAGATAGTGGAGTAGAAGTTTTTCCGGAAACACACCACGGAATAAGTAAGACTCC |
| 85 | TGGTATGAGGAAGATAGTGGAGTAGAAGTTTTTCTTTACAGTTAGCGAACCTCCCGACGTAGGAA |
| 89 | TGGTATGAGGAAGATAGTGGAGTAGAAGTTTTTAGAGGCATAATTTCATCTTCTGACTATAACTA |
| 93 | TGGTATGAGGAAGATAGTGGAGTAGAAGTTTTTGAGCAAAAACTTCTGAATAATGGAAGAAGGAG |
| 97 | TGGTATGAGGAAGATAGTGGAGTAGAAGTTTTTCTAAAGCAAGATAGAACCCTTCTGAATCGTCT |

**Supplementary Table S5. Sequences of staples at complementary to ATTO 488 localized strands**

| | |
|---|---|
| RE-182 | TGGACTCCCTTTTCACCAGTGAGACCTGTCGTTTTTTCGTACTCCAGTCCGAAGTGGCTAT |
| RE-184 | GCCAGCTGCCTGCAGGTCGACTCTGCAAGGCGTTTTTCGTACTCCAGTCCGAAGTGGCTAT |
| RE-186 | ATTAAGTTCGCATCGTAACCGTGCGAGTAACATTTTTCGTACTCCAGTCCGAAGTGGCTAT |
| RE-187 | TAGATGGGGGGTAACGCCAGGGTTGTGCCAAGTTTTTCGTACTCCAGTCCGAAGTGGCTAT |

**Supplementary Table S6. Sequences of staples at sites complementary to biotin modification strands**

| | |
|---|---|
| new204 | ACGTTAGTAAATGAATTTTCTGTAAGCGGAGTGAGAATAGCTTTTGCG |
| new201 | AATAATAATAATAATAATAATAAGTAGAAGTTTTTGGATCGTCGGGTAGCAA CGGCTACTTACTTAGCCGGAACGCTGACCAA |
| new181 | AATAATAATAATAATAATAATAAGTAGAAGTTTTTAGGGCGAAGAACCATCA CCCAAATCAAGTTTTTTGGGGTCAAAGAACG |
| new185 | CTTGCATGCATTAATGAATCGGCCCGCCAGGGTGGTTTTTAACGTCAA |
| new76 | AATAATAATAATAATAATAATAAGTAGAAGTTTTTGATAAGTGAATAGGTGT ATCACCGTACTCAGGAGGTTTAGCGGGGTTT |
| new80 | TGCCTTTAGTCAGACGATTGGCCTGCCAGAATGGAAAGCGACCAGGCG |
| new96 | AATAATAATAATAATAATAATAAGTAGAAGTTTTTCTGAACCTGTTGGCAAA TCAACAGTCATCATATTCCTGATTGATTGTT |
| new99 | GAAATGGATTATTTACATTGGCAGACATTCTGGCCAACAGTCACCTTG |

**Supplementary Table S7. Sequences of strands with special labelling**

| | |
|---|---|
| 3`-biotinDNA | ATTATTATTATTATTATTATTATT - Biotin |
| 5'-ATTO-488 | ATTO 488 - TTTTTATAGCCACTTCGGACTGGAGTACG |
| Or gate out-Cy5 | Cy5-TCCTCTCTCATATACATCTCATCTTCATCATCTTC |
| 5'-Switch-1 | GTGAGGAGTAGAGTGGTATTGGTATG-BHQ2 |
| Switch-2-TET | TET-TCCACTATCTTCCTCATACCAATACCACTCTACTC |
| Switch-2-Cy3 | Cy3-TCCACTATCTTCCTCATACCAATACCACTCTACTC |

**Supplementary Table S8. Sequences of input, R, converter and block**

| | |
|---|---|
| OR gate1-1 | GAGTAGATTGGTGAATAGATGAGATG |
| OR gate-out1 | TCCTCTCTCATATACATCTCATCTATTCACCAATC-Cy5 |
| input-OR gate1 | CATCTCATCTATTCACCAATCTACTC |
| OR gate2-1 | TGTGGGAAGATGATGAAGATGAGATG |
| OR gate-out2 | TCCTCTCTCATATACATCTCATCTTCATCATCTTC-Cy5 |
| input-OR gate2 | CATCTCATCTTCATCATCTTCCCACA |
| Cy5-Block | TCCTCTCTCATATACA |
| R | TCTACTCCTCTCTCATATACA |
| Converter-1 | TGAGATGTATATGAGAGAGGAGTAGA |
| Converter-2-C | CATACCAATACCACTCTACTCCTCACTCATATACA |
| Converter-2- L | CATACCAATACCACTCTACACCTCTCTCATATACA |
| Converter-2-M | CATACCAATACCACTCTACTCCTCACTCATATACA |
| Converter-2-R | CATACCAATACCACTCTACTCCTCTCTCAAATACA |
| Cy3-Block | TCCACTATCTTCCTCA |
